# Quantifying the Effects of Geometric Parameters on the Elastic Properties of Multilayer Graphene Platelet Films

**DOI:** 10.1002/adma.202502546

**Published:** 2025-06-02

**Authors:** Penghao Qi, Xindong Chen, Hanxing Zhu, Yongtao Lyu, Bu Zhang, Qing Peng, Xiqiao Feng, Tongxiang Fan, Di Zhang

**Affiliations:** ^1^ School of Engineering Cardiff University Cardiff CF24 3AA UK; ^2^ Institute of Biomechanics and Medical Engineering, AML, Department of Engineering Mechanics Tsinghua University Beijing 100084 China; ^3^ Department of Engineering Mechanics Dalian University of Technology No. 2 Linggong Road Dalian 116024 China; ^4^ State Key Laboratory of Structural Analysis for Industrial Equipment Dalian University of Technology No. 2 Linggong Road Dalian 116024 China; ^5^ Key Laboratory of Urban Security and Disaster Engineering of Ministry of Education Beijing University of Technology Beijing 100124 China; ^6^ School of Engineering Sciences University of Chinese Academy of Sciences Beijing 100049 China; ^7^ State Key Lab of Metal Matrix Composites Shanghai Jiaotong University Shanghai 200240 China

**Keywords:** defect effects, deformation mechanisms, elastic properties, finite element simulation, multilayer graphene platelet films, size effects

## Abstract

Multilayer graphene platelet films (MGPFs) are widely studied for their exceptional mechanical, electrical, and chemical properties. The elastic properties and deformation mechanisms of MGPFs are highly sensitive to their geometric parameters, including graphene platelet size, graphene area fraction, and layer count. Despite extensive experimental and theoretical efforts, systematically quantifying these effects remains a significant challenge, severely hindering the design of high‐performance MGPFs. Here, realistic random 3D periodic representative volume element (RVE) models of MGPFs are constructed to perform simulations, quantify the effects of different geometric parameters on all their five independent elastic properties, and uncover the dominant deformation mechanisms. The results reveal that the dimensionless platelet size, graphene area fraction, and number of platelet layers significantly affect the elastic properties, with detailed quantifications provided for their relationships. The effects of defects on the elastic properties are also explored, offering insights into the dominant deformation mechanisms. Validation against experimental data confirms that the developed RVE models and dimensionless results apply to various multilayer laminate composites, including MGPFs, MXene, graphene oxide films, and nacre‐like materials. The findings provide a robust framework and pave the way for optimizing the design of MGPFs and other laminate composites, enabling their potential in diverse applications.

## Graphical abstract

Realistic random 3D periodic representative volume element models of multilayer graphene platelet films are constructed to perform simulations, quantify the effects of different geometric parameters on all their five independent elastic properties, and uncover the dominant deformation mechanisms. The obtained dimensionless results apply to different types of laminate or multi‐layered materials such as MXene, graphene oxide films, and nacre and seashells.

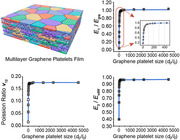

## 1 Introduction

Graphene, a single layer of carbon atoms arranged in a two‐dimensional hexagonal lattice, has emerged as a revolutionary material owing to its exceptional mechanical, electrical, and thermal properties.^[^ ^1^, ^2^ ^]^ Its extraordinary strength, stiffness, and electrical conductivity have positioned monolayer graphene as an ideal candidate for advanced engineering and technological applications, including flexible electronics, energy storage systems, and multifunctional composites.^[^ ^2^, ^3^, ^4^ ^]^ Despite these advantages, large‐scale application of defect‐free and large‐size graphene remains a significant challenge.^[^ ^5^ ^]^ This limitation has shifted focus toward macroscale materials constructed from graphene nanosheets, which are more practical to produce and offer enhanced structural stability and processability while retaining many of graphene's exceptional properties.^[^ ^6^, ^7^ ^]^ These materials—such as graphene‐based membranes, fibers, and composite reinforcements—combine high strength, stiffness, and excellent electrical and thermal conductivity, making them ideal for use in flexible electronics, lightweight structural components, and diverse multifunctional applications.^[^ ^8^, ^9^, ^10^, ^11^, ^12^ ^]^ Moreover, their integration as functional or mechanical components enables diversification of composite material performance, expanding their potential in critical fields.^[^ ^13^ ^]^

Recent advances in fabrication techniques, such as layer‐by‐layer self‐assembly, vacuum‐assisted filtration, and squeegee coating, have enabled the construction of ordered, orientationally aligned, and hierarchically structured graphene materials.^[^ ^14^, ^15^, ^16^, ^17^ ^]^ Among these, bio‐inspired strategies to design nacre‐like layered graphene films with a layer‐by‐layer microstructure have shown particular promise. These materials exhibit a dense, ordered architecture that allows efficient load transmission via interlayer crosslinking, resulting in outstanding mechanical strength, stiffness, and toughness. The practicality of such materials has been well‐proven in recent years. Chen et al.^[^ ^13^ ^]^ demonstrated the use of evaporation‐assisted self‐assembly (ESA) to produce layered graphene‐oxide (GO) films as building blocks for heterogeneous structures with unique mechanical and electrical properties. Similarly, Li et al.^[^ ^18^ ^]^ improved the alignment of MXene nanosheets through a layer‐by‐layer scratch coating process, fabricating ultra‐strong macroscopic films with nacre‐like structures. Yang et al.^[^ ^19^ ^]^ developed a pearl‐layer bionic graphene oxide‐based composite film based on hierarchical structural and interfacial features, which exhibits excellent strength and toughness. Cao et al.^[^ ^20^ ^]^ on the other hand, based on a biomimetic design, used biomass derivatives to significantly improve the flame retardancy and mechanical strength of GO film‐based structural materials, and achieved the desired ultra‐sensitive fire warning response.

Despite these advances, challenges remain in comprehensive understanding of the relationships between the geometrical parameters of multilayer graphene platelet films (MGPFs) and their mechanical properties. In many cases,^[^ ^7^, ^21^, ^22^ ^]^ the performance of fabricated materials falls short of theoretical expectations, largely due to the limited ability of experimental methods to fully characterize the macroscopic geometrical patterns of densely packed graphene assemblies. Current experiments can only probe local structural features, leaving the overall influences of lamellar arrangements and other geometric factors on mechanical properties unclear. This gap in understanding hinders the development of MGPFs with optimized mechanical performance, which is essential for approaching the ideal strength and multifunctional potential of such materials. To address this, computational models offer a promising avenue. Liu et al.^[^ ^23^, ^24^, ^25^ ^]^ developed a series of two‐dimensional tensile‐shear models that provided insights into the mechanical properties of graphene laminates, successfully elucidating their intrinsic mechanical behavior under 2D laminar arrangements. However, such models fall short in capturing the microstructural complexity in other directions within the MGPF layer plane. Similarly, Zhang et al.^[^ ^26^ ^]^ extended the tensile‐shear chain model from a previous study^[^ ^27^ ^]^ to analyze the stiffness and strength properties of staggered laminates with various distribution patterns. Their work, however, was limited only in 2D geometry and unidirectional mechanical properties. The complex multidirectional stress transmission inherent in the 3D structure of MGPF, the variations in irregular platelet size and distribution across different layers, and their interaction effects can't be captured by a two‐dimensional model. Tang et al.^[^ ^28^ ^]^ proposed an interfacial model to describe the elastic‐viscoelastic behavior of nacre‐like laminate materials and analyzed reinforcement mechanisms based on experimental data. Nevertheless, their work did not extend to examining the effects of lamellar arrangement structure or other geometric parameters on the mechanical properties.

To the best of our knowledge, nobody has employed the multilayer random Voronoi graphene platelet model to simulate the mechanical properties of MGPFs and to investigate how their mechanical properties depend on the geometric parameters of the graphene platelets. This work for the first time uses 3D multilayer random periodic Voronoi graphene platelet models to obtain all the five independent elastic constants of MGPFs using finite element simulation and to quantify the effects of the different geometric parameters and the number of graphene platelet layers on these five independent elastic constants. All the obtained results are normalized, making them applicable in different types of laminated materials. This work not only establishes a robust framework for understanding and optimizing the design and fabrication of MGPFs with tailored and desired mechanical properties but also provides important and practical guidance for the rational design of different types of macroscopic laminated composites such as nacre‐like laminates.

## 2 Geometric Model and Finite Element Treatment

### 2.1 Construction of Geometric Model of MGPFs

In this work, 3D random periodic representative volume elements (RVEs) are constructed to simulate the elastic properties of MGPFs. In the RVEs, each layer of the MGPFs is represented by a periodic 2D square random Voronoi platelet model containing *N* complete graphene platelets, as shown in Figure S1 (Supporting Information), where the solid lines represent the boundary gaps between the intralayer neighboring graphene platelets. If the side length of the 2D Voronoi models (i.e., each layer of the MGPFs) is *L* and all the graphene platelets are identical regular hexagons, the distance *d_0_* between the centers of two intralayer neighboring graphene platelets can be obtained as:^[^ ^29^, ^30^ ^]^

(1) $$d_{\circ }=\frac{2L}{\sqrt{2\sqrt{3}N}}$$

To construct a random Voronoi graphene platelet model with *N* complete platelets, if the smallest distance between the centers of any two interlayer neighboring graphene platelets is *δ*, the regularity degree *α* of the random Voronoi (Tessellation) model was first defined by Zhu et al.^[^ ^29^, ^30^ ^]^ and given as:

(2) $$\alpha =\frac{\delta }{d_{\circ }}$$

If *α* = 1, all platelets are identical regular hexagons (Figure S1f, Supporting Information), while α = 0 represents completely random irregular polygons with 3–11 sides.^[^ ^29^ ^]^

**Figure 1:**
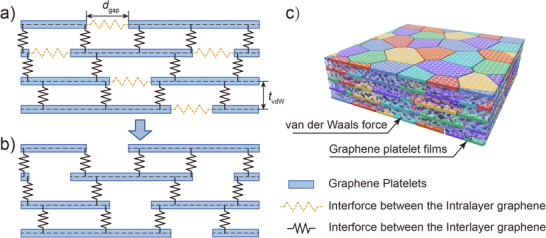
Schematic representation of the overall structure of MGPFs and the interlayer interaction of graphene sheets. a) Inclusive of Intralayer Interaction, b) exclusive of Intralayer Interaction, c) 3D periodic random RVE model of MGPFs.

A 3D random periodic representative volume element (RVE) of the multilayer graphene platelet films (MGPFs) is illustrated in **Figure** **1**. Each of the graphene platelet layers is modeled as an independent periodic 2D square random Voronoi structure with uniform geometric characteristics: identical platelet count, regularity, side length, and thickness. For simplicity, all boundary gaps between the intralayer neighboring graphene platelets are assumed to have a uniform width, as depicted by the solid lines in Figure S1 (Supporting Information). These gaps are set to 1 nm or larger, allowing the interaction forces between intralayer platelets, as represented by horizontal yellow springs in Figure 1a, to be neglected.^[^ ^31^, ^32^ ^]^

The graphene platelets within the film are primarily held together by van der Waals forces acting between interlayer staggered graphene platelets, shown in Figure 1b. Adjacent layers are assumed to have a uniform interlayer spacing of h_0_ = 0.34 nm, with the interlayer interaction represented as an equivalent isotropic linear elastic solid material of the same thickness, as shown in Figure 1c. The sliding motion between concentric carbon nanotubes or staggered graphene sheets, governed by van der Waals forces, depends on their relative positions.^[^ ^33^, ^34^, ^35^, ^36^, ^37^ ^]^ Prior studies confirm that the van der Waals force exhibits a linear elastic relationship with sliding deformation under small strain.^[^ ^38^, ^39^, ^40^, ^41^, ^42^ ^]^ Direct mechanical measurements of line tension and friction in mesoscale graphene structures further validate the proportionality between shear force and strain.^[^ ^43^, ^44^ ^]^ Consequently, representing interlayer interactions via an isotropic linear elastic solid layer is appropriate for finite element simulations of MGPFs.

Obviously, the mechanical properties of MGPFs depend on the total overlap area of the staggered graphene platelets, and the latter depends on the area fraction of the graphene platelets in each layer of the MGPFs (or the RVEs), as defined by,

(3) $$F_{A}=\frac{\sum_{\left(i=1\right)}^{N}A_{i}}{L^{2}}$$

where *A*_i_ are the areas of the individual graphene platelets in a layer, *N* is the total number of complete graphene platelets in a layer, and *L* is the in‐plane side length of the RVE model. Evidently, the value of the area fraction *F*_A_ parameter depends upon the regularity degree of the Voronoi graphene platelets and the uniform gap width between the neighboring graphene platelets in the same graphene layer.^[^ ^29^ ^]^ It is noteworthy that, in each RVE model of MGPFs, each of the Voronoi graphene platelet layers has the same geometric parameters and thus statistically has the same value of the *F*_A_ parameter.

### 2.2 Material Properties and Finite Element Treatment of the RVE Model

The commercial finite element software ABAQUS was used to simulate the mechanical properties of MGPFs. The accuracy of the simulation results largely depends on the correct properties of the component materials in the model. Lee et al.^[^ ^2^ ^]^ were the first to experimentally obtain the tensile stiffness of monolayer graphene, 340 N m^−1^, and found that the in‐plane mechanical properties of monolayer graphene are approximately isotropic. Shen and Wu,^[^ ^45^ ^]^ through molecular dynamics simulations, analyzed the effect of interlayer shear on the bending properties of multilayer graphene, and obtained the interlayer shear modulus as 4.6 GPa. Simulation and experimental results^[^ ^45^, ^46^, ^47^ ^]^ indicated that the interlayer shear modulus of multilayer graphene is two to three orders of magnitude lower than the in‐plane Young's modulus, and the interlayer shear plays an important role in the bending deformation behavior.

It has been generally recognized^[^ ^2^, ^47^, ^48^, ^49^ ^]^ that the bending stiffness of monolayer graphene lies in the range of 1.2–1.7 eV (i.e., (1.92  − 2.72) × 10^−19^Nm^−1^), and its tensile stiffness is ≈340 N m^−1^. These parameters are used to determine the mechanical properties of the component materials in the RVE model of multilayer graphene platelet films (MGPFs). In the finite element simulations, the graphene platelets in the RVE model of MGPFs, shown in Figure 1c, are treated as polygonal thin shells of uniform thickness. These shells are assumed to be made of an isotropic linear elastic solid material with a Poisson's ratio of 0.178. To ensure the same out‐of‐plane bending stiffness (2.385 × 10^−19^N m^−1^) and the same in‐plane tensile stiffness (340 *N *m*^−1^*) of the monolayer graphene, the thickness of all the equivalent shell elements in the RVE model of MGPFs is determined as 0.0 9029 nm and the Young's modulus of the equivalent isotropic shell solid material is obtained as 3765.6 GPa, more details can be found in supplementary document. The graphene platelets in each layer of the RVE models are meshed into at least 15 000 four‐noded SC4R shell elements.

Due to its highly anisotropic, layered structure, graphene exhibits a large difference between its in‐plane and out‐of‐plane stiffnesses. This characteristic enables graphene to be exfoliated into single‐layer nanosheets without damaging its in‐plane structure.^[^ ^14^, ^15^, ^16^ ^]^ Experimentally, the interlayer binding energy has been measured to be ≈0.25 J m^−^^2^,^[^ ^34^ ^]^ while the in‐plane stiffness is ≈340 N m^−1^. These values suggest that the interlayer (i.e., the out‐of‐plane) mechanical properties are at least 2–3 orders of magnitude lower than the in‐plane properties.^[^ ^34^, ^45^, ^47^ ^]^ In this model, the uniform thickness of the van der Waals interaction layer is set to 0.34 nm. The van der Waals interaction between any two staggered neighboring graphene platelets is represented by a thin layer of equivalent isotropic linear elastic solid material with Young's modulus of 10 GPa (E_vdW_ = 10 GPa), a Poisson's ratio of 0.001 (ν_vdW_ = 0.001) and the same dimensions as the overlap area. The shear modulus of this equivalent isotropic solid layer material is ≈5 GPa (G_vdW_ = 5 GPa), and consistent with those in theoretical analyses, molecular dynamics simulations, and experimental measurements.^[^ ^45^, ^47^, ^50^ ^]^ As the in‐plane dimensions of the overlap region between staggered graphene platelets (≈100 nm) are significantly larger than the interlayer spacing (0.34 nm), the equivalent solid layer for the van der Waals interaction is meshed into large number of single‐layer C3D8R solid elements. Thus, the top and bottom surfaces of these solid elements share nodes with the shell elements (S4R) of the staggered graphene platelets in the RVE model. Despite the overlap of these two types of elements in the 3D space of the RVE model, ABAQUS finite element simulations can handle this without issue, ensuring an accurate representation of the mechanical behavior of the MGPFs.

In finite element simulations, periodic boundary conditions are consistently applied to the corresponding nodes on the side faces of the RVE models. When the number of graphene platelet layers exceeds 10, the 2D square periodic random Voronoi structure of the top layer in the RVE model is made identical to that of the bottom layer. Consequently, periodic boundary conditions are also applied to the corresponding nodes on the top and bottom layers during simulations. A small tensile or shear strain is applied to the RVE models to determine the in‐plane or out‐of‐plane Young's modulus, Poisson's ratio, or shear modulus of the MGPFs. It is worth noting that each RVE model may contain over one billion atoms and thousands of simulations are included in this work, thus, it is not feasible to use atomistic simulation to obtain the results in this work.

## 3 Quantifying the Effects of Different Geometric Parameters

Each layer of the RVE models should include a sufficient number of complete graphene platelets to ensure that the properties derived from the individual RVE models are stable and in‐plane isotropic. However, the total computational cost must remain feasible, as thousands of simulations are required to generate all the data for this study. Based on the results presented in Table S1 (Supporting Information) the number of complete graphene platelets in each layer of the RVE models is fixed at N = 100 for this work. Since the mechanical properties of MGPFs are obviously in‐plane isotropic (i.e., *E*_1_ =  2*G*_12_(1 + *v*_12_), see Table S2 Supporting Information) and MGPFs have three orthogonal planes of elastic symmetry, they have only five independent elastic constants, i.e., *E*_1_, *v*_12_, *E*_3_, *v*_31_ and *G*_31_, to be determined.^[^ ^51^ ^]^ In this work, the effects of the most significant geometric parameters on all these five independent elastic properties of MGPFs are presented in the main text. Each data point in the figures represents the mean result obtained from 20 random RVE models with the same combination of different geometric parameters, as illustrated in Tables S2 and S3 (Supporting Information). Additionally, the effects of other parameters, such as degree of graphene platelet regularity, are illustrated in the supplementary material.

### 3.1 Effects of the Mean Size of Graphene Platelets

When the MGPFs are in‐plane stretched, the applied tensile force is transmitted via the interlayer shear stress resulted from the van der Waals interactions between the staggered graphene platelets.^[^ ^25^, ^38^ ^]^ Liu et al.^[^ ^25^ ^]^ analyzed the unidirectional mechanical properties of MGPFs based on a regular 2D tension‐shear chain model in which the graphene platelets were represented by 1D bars with the same length *l* and the same orientation, and all the overlaps between the regularly staggered graphene platelets were assumed to be the same as *l*/2. A characteristic platelet length^[^ ^25^ ^]^ to characterize the length scale of effective interlayer load transmission was obtained as $l_{0}=\sqrt{Dh_{0}/\left(4G_{vdW}\right)}=2.404nm,$ where *D* is the in‐plane tensile stiffness (i.e., 340 *N *m^−1^) of the graphene platelets, *h*_0_ is the interlayer distance between the staggered neighboring graphene platelets (i.e., the thickness of van der Waals interaction layer 0.34 nm), and *G_vdW_* is the shear modulus (i.e., 5 GPa) of the equivalent elastic solid material of the van der Waals interaction layer. The unidirectional mechanical properties^[^ ^25^, ^26^ ^]^ of MGPFs were found to be significantly dependent on the length *l* of the graphene platelets if *l* is close to *l*_0_ or smaller, and become less dependent on the length of the graphene platelets if *l* is several times larger than *l*_0_. However, if the lengths of the platelets are the same but they are randomly staggered, the unidirectional mechanical properties of the platelet material obtained from a random 2D model^[^ ^26^ ^]^ were found to be more sensitive to the length.

**Figure 2:**
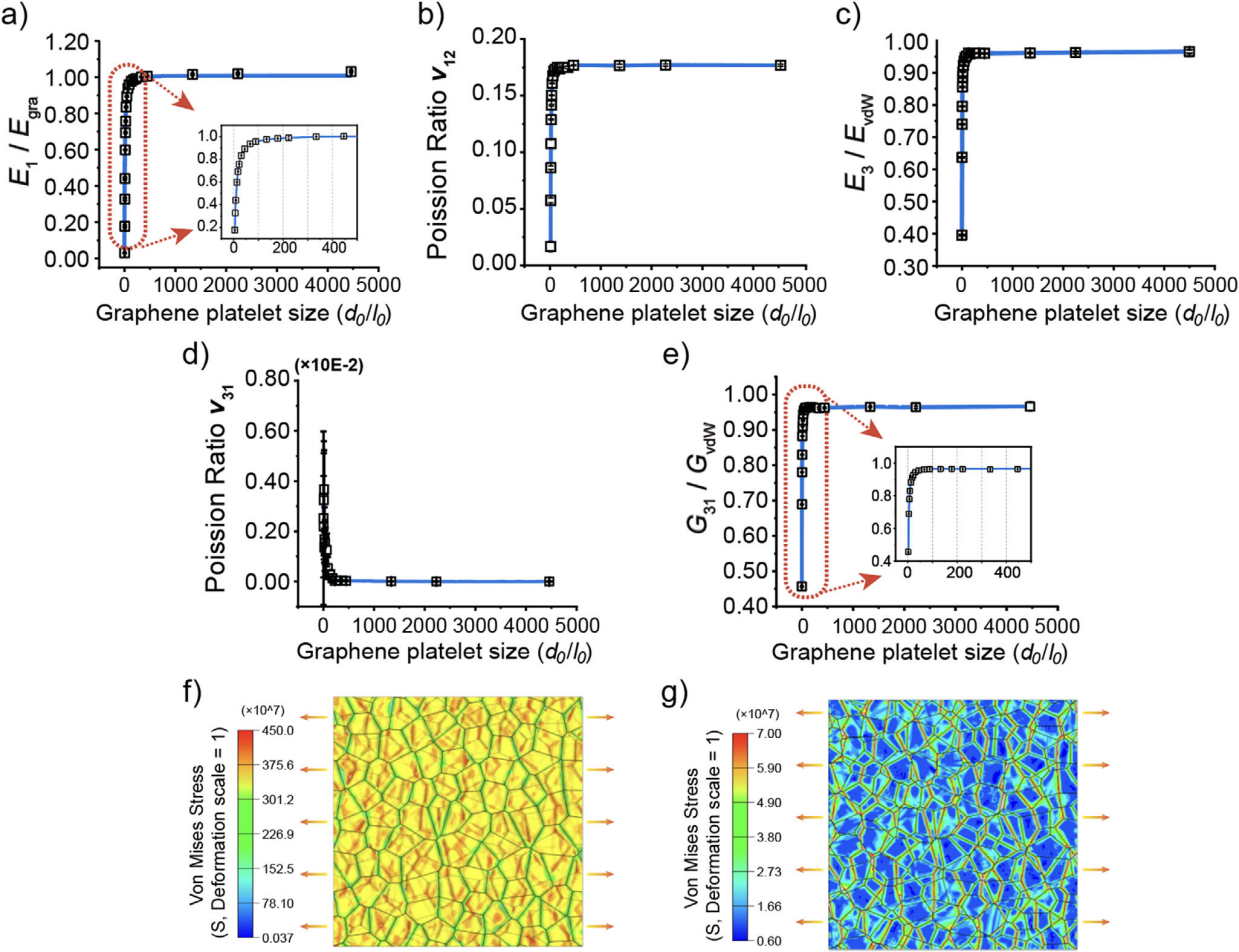
Effects of dimensionless mean graphene platelet size (*d*_0_/*l*_0_) on the elastic properties of MGPFs. RVE models with the number of graphene layers *M* = 5, the regularity degree *α* = 0.6, and a fixed uniform gap of 1 nm between the intralayer neighboring graphene platelets. a) dimensionless in‐plane Young's modulus $\bar{E_{1}}$, b) in‐plane Poisson's ratio *v*_12_, c) dimensionless out‐of‐plane Young's modulus $\bar{E_{3}}$, d) out‐of‐plane Poisson's ratio *v*_31_, e) dimensionless out‐of‐plane shear modulus $\bar{G_{31}}$, f) and g) von Mises stress contours in the graphene platelets and in the equivalent elastic solid layer material for the van der Waals interaction between the staggered graphene platelets, respectively, when the MGPF with *d*_0_/*l*_0_ = 44.64 is under a uniaxial in‐plane tensile strain of 0.001.

**Figure** **2** shows the effects of the graphene platelet dimensionless mean size (*d*_0_/*l*_0_) on the elastic properties of MGPFs with the number of graphene layers fixed at *M* = 5, the regularity degree fixed at *α* = 0.6, and a fixed uniform gap of 1 nm between the intralayer neighboring graphene platelets, where the in‐plane Young's modulus is normalized by that of the perfect graphene (i.e., *E_gra_* =  1000 GPa), the out‐of‐plane Young's modulus is normalized by the Young's modulus of the equivalent isotropic solid layer material for van der Waals interaction (i.e., *E_vdW_* = 10 GPa), and the out‐of‐plane shear modulus is normalized by the shear modulus of the equivalent isotropic solid layer material (i.e., *G_vdW_* = 5 GPa), respectively. Both the in‐plane stiffness (Figure 2a) and Poisson's ratio (Figure 2b) of MGPFs are very sensitive to the value of *d*_0_/*l*_0_, increase rapidly with the increase of *d*_0_/*l*_0_ when the value of *d*_0_/*l*_0_ is small, then their increases slow down with the further increase of *d*_0_/*l*_0_, and finally their values approach the Young's modulus *E_gra_* and Poisson's ratio *v_gra_* (i.e., 0.178) of the perfect graphene sheet when the value of *d*_0_/*l*_0_ is sufficiently large (e.g., *d*_0_/ *l*_0_ =  1000). This is because when the MGPFs are in‐plane stretched, their stiffness and lateral deformation are mainly dependent on those of the equivalent solid layer material for the van der Waals interaction between the staggered graphene platelets if *d*_0_/*l*_0_ is small, resulting in small values of the in‐plane Young's modulus and Poisson's ratio of MGPFs, and their strong dependences on *d*_0_/*l*_0_. However, their in‐plane stiffness and deformation become dominated by the in‐plane Young's modulus and Poisson's ratio of the graphene platelets if *d*_0_/*l*_0_ is sufficiently large, making the in‐plane Young's modulus and Poisson's ratio of MGPFs almost the same as those of the perfect monolayer graphene sheet.

Figure 2c,e illustrates that the out‐of‐plane Young's modulus *E*_3_ and shear modulus *G*_31_ of MGPFs depend on not only the Young's modulus and shear modulus of the equivalent solid layer material for van der Waals interaction, but also the graphene overlap ratio between the staggered neighboring graphene platelet layers, making the out‐of‐plane stiffnesses of MGPFs increase rapidly with the increase of *d*_0_/*l*_0_ when the value of *d*_0_/*l*_0_ is small (this is because the graphene overlap ratio is more sensitive to *d*_0_/*l*_0_ when its value is small). With the further increase of *d*_0_/*l*_0_, their increases slow down, and their values approach those of the equivalent solid layer material for van der Waals interaction when the value of *d*_0_/*l*_0_ is sufficiently large (e.g., *d*_0_/ *l*_0_ =  2000). As the Poisson's ratio of the equivalent solid material for van der Waals interaction is very small (assumed to be 0.001 in finite element treatment) and the much stiffer graphene platelets significantly restrain the lateral deformation of MGPFs when the MGPFs are deformed in the out‐of‐plane directions, which makes the out‐of‐plane Poisson's ratio *v*_31_ of MGPFs very close to 0 as can be seen in Figure 2d. When *d*_0_/*l*_0_ is very small, the slightly larger value of *v*_31_ may be caused by mesh sensitivity. When an MGPF with *d*_0_/ *l*_0_ = 44.64 is in‐plane uniaxially stretched to a strain of 0.001, von Mises stress concentration occurs at the edges of the overlap areas between the staggered graphene platelets as can be seen in Figure 2f,g, which is consistent with the theoretical results.^[^ ^25^ ^]^ In addition, Figure 2f,g demonstrates clearly that the stress magnitude in the graphene platelets is much larger than that in the equivalent elastic solid layer material for the van der Waals interactions.

If the dimensionless mean size *d*_0_/ *l*_0_ of the graphene platelets tends to infinite, the values of the in‐plane Young's modulus *E*_1_ of MGPFs predicted by either our 3D model or by the 2D regular^[^ ^25^ ^]^ and random^[^ ^26^ ^]^ models will become the same as the value (i.e., *E_gra_* =  1000 GPa) of the perfect monolayer graphene sheet. **Table** **1** demonstrates how the values of the in‐plane Young's modulus. *E*_1_ of MGPFs predicted by the 2D models^[^ ^25^, ^26^ ^]^ and our 3D model depend on the value of *d*_0_/ *l*_0_, where our 3D RVE model has fixed parameters of *M* = 5, *α* = 0.6, and a fixed uniform gap of 1 nm between the intralayer neighboring graphene platelets. For the obtained value of *E*_1_ to reach 80% or 90% of the maximum value (i.e., *E_gra_* =  1000 GPa), our 3D RVE model always requires a much larger value of *d*_0_/ *l*_0_ than the 2D models, indicating that the stiffness obtained from our realistic 3D random model is more sensitive to *d*_0_/ *l*_0_ than the 2D regular and random models.^[^ ^25^, ^26^ ^]^ This is because our realistic 3D model has a much larger irregularity or randomness than the 2D random model,^[^ ^25^, ^26^ ^]^ and moreover, the 2D regular model^[^ ^25^ ^]^ does not contain any randomness or irregularity at all.

**Table 1:** The mean dimensionless size (diameter) *d*_0_/*l*_0_ of graphene platelets required for the in‐plane stiffness *E*_1_ obtained from our 3D RVE model of MGPFs, or the 2D regular^[^ ^25^ ^]^ or random^[^ ^26^ ^]^ models to reach 80% or 90% the Young's modulus (i.e., 1000 GPa) of the perfect graphene.

Model	80%	90%
2D regular model^[^ ^25^ ^]^	8	18
2D random model^[^ ^26^ ^]^	15.9	23.9
Our 3D random model	26.5	47.5

### 3.2 Effects of the Area Fraction *F_A_* of Graphene Platelets

**Figure 3:**
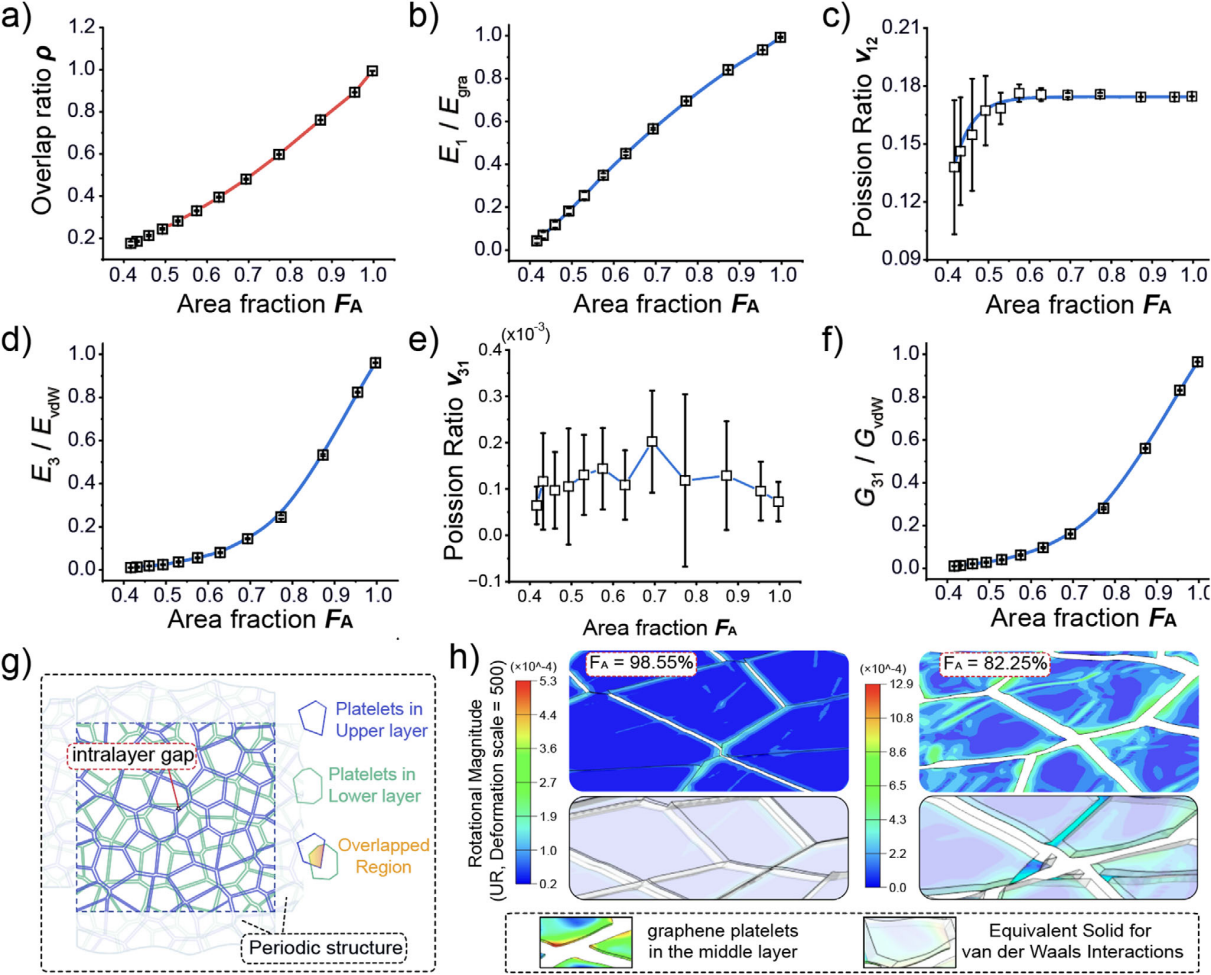
Effects of the graphene area fraction *F_A_* on the elastic properties of MGPFs. RVE model with a mean graphene diameter *d*_0_ = 500 nm, number of graphene layers *M* = 5 and graphene platelet regularity *α* = 0.6. a) graphene area fraction *F_A_* versus graphene overlap ratio *ρ*, b) dimensionless in‐plane Young's modulus $\bar{E_{1}}$, c) in‐plane Poisson's ratio *v*_12_, d) dimensionless out‐of‐plane Young's modulus $\bar{E_{3}}$, e) out‐of‐plane Poisson's ratio *v*_31_, f) dimensionless out‐of‐plane shear modulus $\bar{G_{31}}$, g) schematic of the graphene platelets in two neighboring layers of a periodic RVE model, and the overlap area of two staggered graphene platelets. h) Comparison of the rotation magnitudes of the graphene platelets in the middle layer of RVE models with different values of the graphene area fraction *F_A_* under the same shear boundary condition.

As all the individual graphene platelets are held together by the van der Waals interaction forces, both the in‐plane and out‐of‐plane stiffnesses of the MGPFs depend significantly on the graphene area fraction *F_A_* and the graphene overlap ratio *ρ*. **Figure** **3**a shows clearly that the graphene overlap ratio *ρ*, defined as the total overlap area between the graphene platelets in the two neighboring layers in the RVE model divided by the model in‐plane area *L* ^2^, is a quadratic function of the graphene area fraction *F_A_*, i.e., $\rho \propto F_{A}^{2}$. When *F_A_* is small, the in‐plane Young's modulus *E*_1_ is approximately proportional to *F_A_* or the square root of *ρ*, i.e., *E*_1_∝ρ^1/2^∝*F_A_*, as can be seen in Figure 3b. With the increase of *F_A_*, the increase of *E*_1_ slows down and the value of *E*_1_ gradually approaches its maximum value (e.g., 98% of *E_gra_* when *F_A_* =  99.6%). The results in Figure S2 (Supporting Information) demonstrate that when an MGPF is in‐plane stretched, the smaller the graphene area fraction *F_A_*, the larger the magnitude of the von Mises stress, and the more uneven of the stress distribution in different graphene platelets. Figure 3c indicates that the in‐plane Poisson's ratio *v*_12_ increases quickly with *F_A_* when *F_A_* is small, and then approaches a stable value when *F_A_* is larger than 0.6. This is because the smaller the value of *F_A_*, the smaller the overlap ratio ρ, consequently the value of *v*_12_ depends more on the deformation of the equivalent elastic solid layer material for van der Waals interactions, resulting in smaller value of *v*_12_ when *F_A_* is small. When *F_A_* (or *ρ*) is large, the lateral deformation of the MGPF mainly depends on that of the graphene platelets, making the in‐plane Poisson's ratio almost the same as *v_gra_* (i.e., 0.178). The results in Figure 3d,f exhibit clearly that both the out‐of‐plane Young's modulus *E*_3_ and shear modulus *G*_31_ of MGPFs are small when *F_A_* is small, and increase very quickly with the increment of  *F_A_* when *F_A_* is larger than 0.75. However, the out‐of‐plane Poisson's ratio *v*_31_ of MGPFs shown in Figure 3e is always nearly 0 for different values of *F_A_* due to the same reason explained for the results in Figure 2d. The contour images in Figure S3 (Supporting Information) also demonstrate that when RVE models with different values of *F_A_* are under the same magnitude of in‐plane or out‐of‐plane strain, the smaller the value of *F_A_*, the more easily the graphene platelets to rotate, and thus the smaller the in‐plane and out‐of‐plane stiffnesses of the MGPFs.

The stiffnesses of MGPFs shown in Figure 3b,d,f is normalized by *E_gra_*, *E_vdW_* or *G_vdW_*, respectively. The out‐of‐plane stiffnesses *E*_3_ and *G*_31_ can well be described by a power function of the graphene area fraction *F_A_*, e.g. $E_{3}\propto F_{A}^{k}\;\mathrm{or}\;G_{31}\propto \rho^{2.5}\propto F_{A}^{5}$. For MGPFs with *d*_0_ =  500 nm, α  =  0.6 and different numbers of graphene platelet layers *M*, the powers k can be determined from their log–log plots according to the relevant results as shown in Figure 3d,f, and are presented in Table S4 (Supporting Information), indicating that the larger the number M of the graphene platelet layers, the larger the value of k in the power function of $F_{A}^{k}.$ Figure 3g is a schematic of the graphene platelets in two neighboring layers of a periodic RVE model, and the overlap area of two staggered graphene platelets. The contour diagrams of the rotational magnitudes of the graphene platelets in the middle layer of RVE models, shown in Figure 3h, demonstrate clearly that when the RVEs are deformed under the same shear boundary condition, the smaller the value of *F_A_*, the larger the rotational magnitude of the graphene platelets in the middle layer, i.e., the smaller the shear modulus of the MGPF. The contour diagrams in Figure S3 (Supporting Information) also clearly support the above results.

### 3.3 Effects of the Number *M* of Graphene Platelet Layers

**Figure 4:**
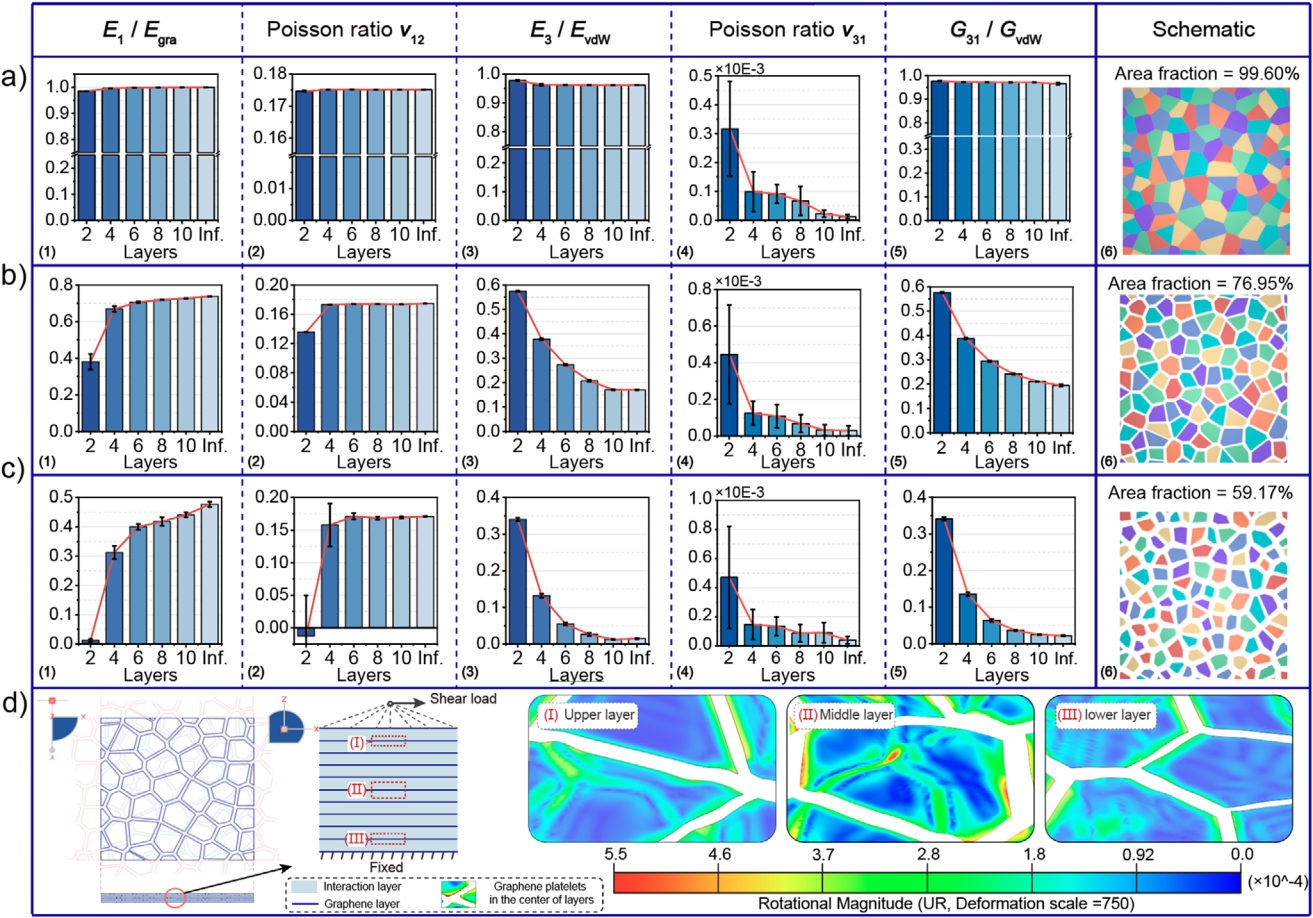
Effects of the number *M* of graphene layers on the dimensionless elastic properties of MGPFs. RVE model with a fixed number of graphene platelets *N* = 100, a fixed graphene platelet mean size *d*_0_ =  500nm, and a graphene regularity degree α = 0.6. Dimensionless elastic properties and geometric structure schematic of MGPFs with (a) area fraction *F_A_* =  99.60%, b) area fraction *F_A_* =  76.95%, c) area fraction *F_A_* =  59.17%. d) Comparison of the rotation magnitudes of the graphene platelets in different layers of the same RVE model under the same type shear boundary condition.

The effects of the number *M* on all the five independent elastic properties of MGPFs with a fixed number of complete graphene platelets *N* = 100, a fixed *d*_0_ =  500 nm, a constant *α* = 0.6 and different values of *F_A_* are presented in **Figure** **4**, where the in‐plane and out‐of‐plane stiffnesses are normalized by *E_gra_*, *E*_*vdW*,_ and *G_vdW_*, respectively. When *F*_A_ = 99.6%, the results in Figure 4a indicate that with the increase of *M*, the in‐plane Young's modulus *E*_1_ and Poisson's ratio *v*_12_ of MGPFs increase slightly and then approach the corresponding values of the perfect monolayer graphene sheet. The out‐of‐plane Young's modulus *E*_3_ and shear modulus *G*_31_ decrease slightly and then approach a stable value with the increase of *M* and remain almost unchanged when *M* ≥ 10. In addition, the out‐of‐plane Poisson's ratio is always very close to that (i.e., 0.001) of the equivalent solid layer material for van der Waals interaction, which dominates the deformation of MGPF when deformed in the thickness direction. The slightly larger value of *v*_31_ when *M* = 2 may be resulted from mesh sensitivity. When the graphene area fractions are *F*_A_ = 76.95% and *F*_A_ = 59.17%, both the in‐plane Young's modulus and Poisson's ratio of MGPFs increase with the increment of *M* and approach a stable value. In contrast, the out‐of‐plane Young's modulus and shear modulus of MGPFs decrease with the increase of *M* and approach a stable value, as shown in Figure 4b (3) and (5) and c (3) and (5). The value of *v*_31_ is always very close to Poisson's ratio of the equivalent solid layer material of the van der Waals interaction. The contour diagrams in Figure 4d show the comparison between the rotational magnitudes of graphene platelets in different graphene layers of the same RVE model when it is deformed under the same shear boundary condition, which indicates that the graphene platelets in the middle layers can more easily rotate than those close to the top or bottom layers, implying that the larger the number of graphene platelet layers *M*, the smaller the out‐of‐plane stiffness of the MGPF. In addition, the contour diagrams in Figure S4 (Supporting Information) demonstrate that when RVE models with the same *F_A_* = 0.591 and different numbers of graphene platelet layers *M* are under the same out‐of‐plane compressive or shear stress, the larger the number of graphene platelet layers *M*, the larger the rotational magnitude of graphene platelets in the middle layer of the RVE model. The contour results in Figure S4 (Supporting Information) indicate clearly that for MGPFs with *F_A_* much smaller than 1, the larger the number of graphene platelet layers *M*, the smaller the out‐of‐plane Young's modulus and shear modulus, clearly supporting the relevant results in Figure 3.

### 3.4 Effects of Defects on the Elastic Properties of MGPFs

**Figure 5:**
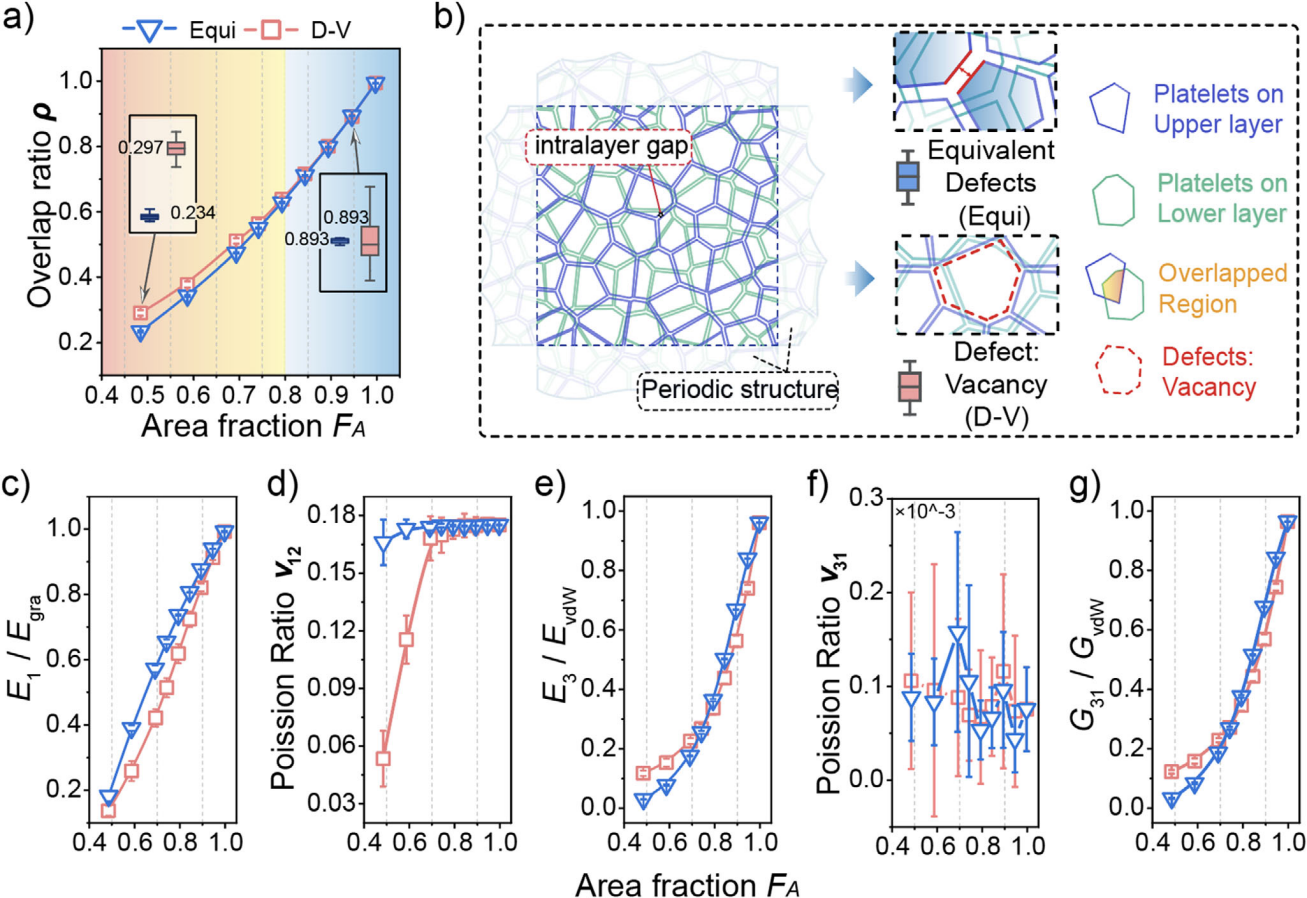
Effects of graphene area fraction *F_A_* (or real defect level 1 − *F_A_*) on the elastic properties of MGPFs with mean graphene diameter *d*_0_ = 500 nm, number of graphene layers *M* = 5 and graphene platelet regularity *α* = 0.6. The relevant results in Figure 3 are included for comparison. a) graphene overlap ratio ρ, b) geometric structure of RVE models with the two types and the same level of defects 1 −  *F_A_* =  27.95%, c) dimensionless in‐plane Young's modulus $\bar{E_{1}}$, d) in‐plane Poisson's ratio *v*_12_, e) dimensionless out‐of‐plane Young's modulus $\bar{E_{3}}$, f) out‐of‐plane Poisson's ratio *v*_31_, g) dimensionless out‐of‐plane shear modulus $\bar{G_{31}}$.

For a MGPF with a given value of the graphene area fraction *F_A_*, the equivalent level of defects compared to a large size perfect graphene sheet could be quantified as 1 − *F_A_*. In a real MGPF, its defects may be resulted from some missing graphene platelets, which are real concentrated defects. To study the effects of missing graphene platelets (or real concentrated defects) on the elastic properties of MGPFs, RVE models with *M*  =  5, *d*_0_ =  500 nm, α  =  0.6, *L*  =  4.662 µm and a uniform gap of 1 nm between the intralayer neighboring graphene platelets are constructed first. The initial defect level of these initial models is 1 −  *F_A_* =  1 − 99.6%  =  0.4%, which is negligible. According to the results in Figure 3, the elastic properties of these MGPFs with *F_A_* =  99.6% are almost the same as those of perfect large size multilayer graphene platelet sheets. For MGPFs with a given level of real defects 1 − *F_A_*, the corresponding RVE models can be generated by randomly removing some graphene platelets from each layer of the initial RVE models. **Figure** **5** shows the effects of real defects (i.e., missing graphene platelets) on the elastic properties of MGPFs, and the relevant results from Figure 3 (i.e., the effects of the graphene area fraction *F_A_*, or the equivalent defect level 1 − *F_A_*) are also included for comparison, where the stiffnesses of MGPFs are normalized by *E_gra_*, *E_vdW_*, and *G_vdW_*, respectively.

Figure 5a shows that for the same value of graphene area fraction *F_A_* (or the same level of defects 1 − *F_A_*), the graphene overlap ratio ρ of MGPFs with real defects is always larger than or equal to that of the MGPFs with equivalent defects. When the graphene area fraction increases from 0.5 to 0.75, the difference between the graphene overlap ratios of MGPFs with the two types of defects reduces from 25% to nearly 0. Figure 5b illustrates the geometric structures of RVE models with the two types and the same level of defects 1 −  *F_A_* =  27.95%. For the same value of graphene area fraction *F_A_*, Figure 5c,d demonstrate that the in‐plane elastic properties *E*_1_ and *v*_12_ of MGPFs with uniform equivalent defects are always larger than or equal to those of the MGPFs with the same level of real concentrated defects. Their magnitude relative differences are quite large when *F_A_* is 0.4 < *F_A_* < 0.7, reduce with the increase of *F_A_* and gradually approach 0 when *F_A_* ≥ 0.8. In contrast, the out‐of‐plane Young's modulus *E*_3_ (Figure 5e) and shear modulus *G*_31_ (Figure 5g) of MGPFs with real concentrated defects are larger than those of MGPFs with the same level of equivalent defects when 0.5 < *F_A_* < 0.7, and become smaller than those of the latter when 0.75 < *F_A_* < 0.95. The reason may be because the out‐of‐plane stiffnesses depend more on the graphene overlap ratio (see Figure 5a; Table S4, Supporting Information). The magnitude of the out‐of‐plane Poisson's ratio is always very close to zero as shown in Figure 5f.

## 4 Applicability and Generalizability Analysis of the Model and Results

In the mechanics point of view, the RVE model developed for MGPFs in this study can represent a wide range of different two‐phase laminate composite materials. One of the main characteristics of the RVE model of MGPFs (or a kind of composites) is that Young's modulus of the stiffer phase material (i.e., the graphene platelets) is 2–3 orders larger than that of the softer phase material (i.e., the equivalent elastic solid layer material for the van der Waals interaction between the staggered graphene platelets). The obtained in‐plane Young's modulus of MGPFs is normalized by that of the stiffer phase material (i.e., the monolayer perfect graphene), and the obtained out‐of‐plane Young's modulus and shear modulus of MGPFs are normalized by those of the softer phase material. The RVE model developed in this study can effectively capture the complex mechanical properties and accurately reveal the dominant deformation mechanisms of MGPFs (or two‐phase laminate composites), as well as offer wide versatility. The geometric model and the obtained results in this work can be directly extended to applications in similar single‐material systems, such as multilayer graphene oxide and MXene.^[^ ^52^, ^53^ ^]^ Moreover, by adjusting the model parameters related to the elastic properties of the constituent materials (e.g., *E*_vdw_, *G*_vdw_ in this paper), layer thicknesses, and feature sizes, the results obtained in this work can also be used to predict the mechanical properties of other two‐phase laminate composites composed of stiff and soft components, e.g. GO/Graphene‐based composites and real nacre or seashell materials. This is because for such two‐phase laminate composites composed of stiff and soft components, their in‐plane Young's modulus is dominated by that of the stiffer phase material and their out‐of‐plane Young's modulus and shear modulus mainly depend on those of the softer phase material.

**Figure 6:**
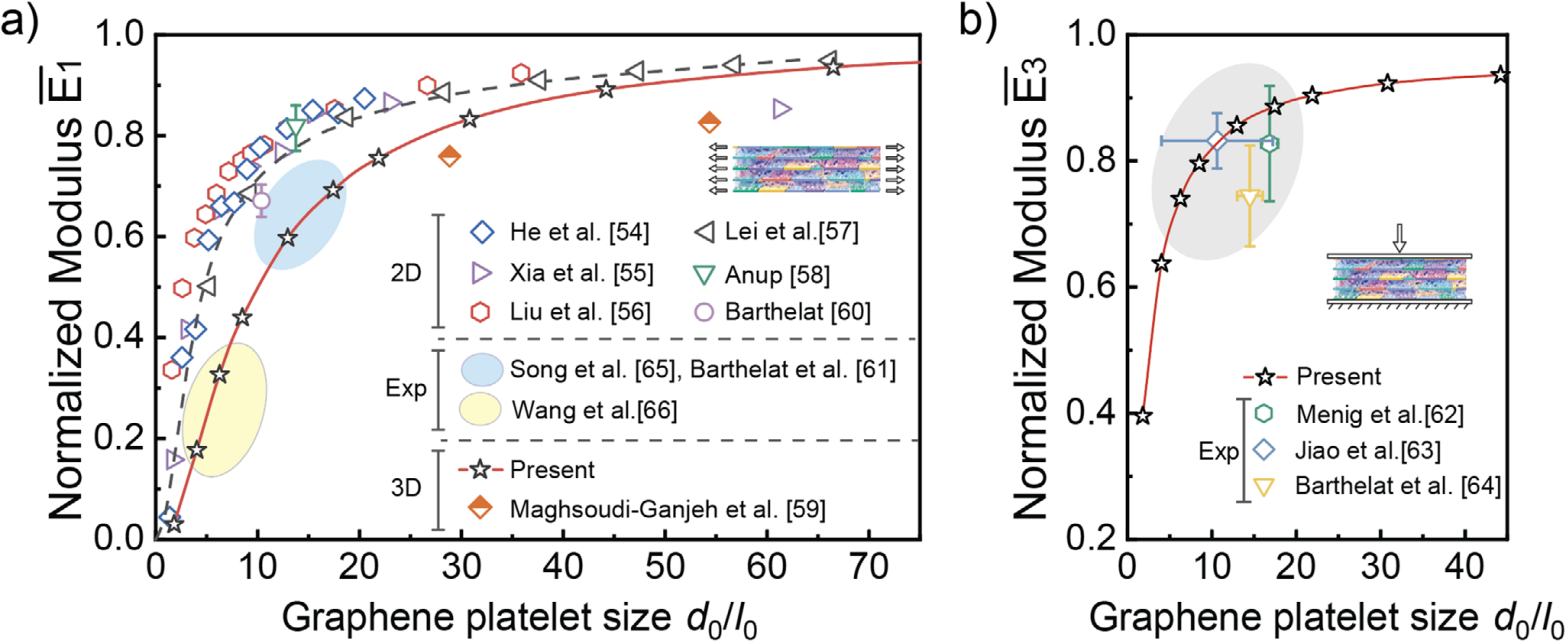
Cross‐application validation of the present simulation results with the experimental and simulation results reported in literature data. a) Dimensionless in‐plane Young's modules and b) Dimensionless out‐of‐plane Young's modulus of different nacre‐like materials as a function of the dimensionless size of the platelet size/length *d*_0_/*l*_0_. Part of data obtained by fitting the linear stage of stress‐strain curves by Python code. The materials from literature include graphene oxide (GO) layered materials based on molecular dynamics method (MD)^[^ ^54^ ^]^ and numerical calculation (NC),^[^ ^54^ ^]^ multilayer graphene assemblies (MLGs),^[^ ^55^ ^]^ multilayer phosphorene assemblies (MLPs),^[^ ^56^ ^]^ nacre‐like bio‐composite,^[^ ^57^, ^58^, ^59^ ^]^ real nacre materials,^[^ ^38^, ^60^, ^61^, ^62^, ^63^, ^64^, ^65^ ^]^ and montmorillonite/poly(vinyl alcohol) (MMT/PVA) composite materials.^[^ ^66^ ^]^

To demonstrate the versatile application of the dimensionless results obtained in this work, the in‐plane and out‐of‐plane Young's moduli shown in Figure 2 are compared with the relevant simulation and experimental results of two‐phase laminate composites in literature as given in Table S5 (Supporting Information). In a typical two‐phase laminate composite, the stiffer laminate is assumed to have a size *d*_0_, a thickness *t_S_* and a Young's modulus *E_S_*; the softer laminate is assumed to have a thickness *t_m_*, a Young's modulus *E_m_* and a shear modulus *G_m_*. The critical size and the dimensionless size of the stiffer laminate can be determined as $l_{0}=\sqrt{E_{S}t_{S}t_{m}/\left(4G_{m}\right)}$ and *d*_0_/*l*_0_. If the in‐plane and out‐of‐plane moduli of such a two‐phase laminate composite are *E*_1_, *E*_3_ and *G*_31_, its dimensionless in‐plane and out‐of‐plane moduli can be approximately obtained as *E*_1_(*t_S_* + *t_m_*)/(*E_S_t_S_*), *E*_3_ *t_m_*/[*E_m_* (*t_S_* + *t_m_*)] and *G*_31_ *t_m_*/[*G_m_* (*t_S_* + *t_m_*)], respectively. Thus, according to the material properties and geometric dimensions of the relevant two‐phase laminate composites in literature, the dimensionless moduli of these composites and the dimensionless size *d*_0_/*l*_0_ of their stiffer laminate can be obtained and presented in Table S5 (Supporting Information). To demonstrate the versatile application of the results obtained in this work, the dimensionless in‐plane and out‐of‐plane Young's moduli of the different laminate composites in literature (as shown in Table S5, Supporting Information) are plotted in **Figure** **6**a,b, and our relevant results in Figure 2 are also included for comparison. As can be seen in Figure 6a,b, the relevant results in literature are well in line with our simulation results obtained in this work, clearly confirming the versatile applications of the results obtained in this work. The results in Figure 6a indicate that most of the dimensionless in‐plane Young's moduli reported in literature could get close to 1.0 at a smaller value of *d*_0_/*l*_0_ than our results, this may be because our geometric model contains more randomness and irregularity than the models or experimental materials reported in literature, as demonstrated in Table 1.

## 5 Conclusion

The periodic 3D random RVE model developed in this work can well capture the geometric characteristics of MGPFs and accurately reveal their dominant deformation mechanisms. Moreover, the constructed REV model and the obtained dimensionless results can be extended to applications in a wide range of different types of laminate or multi‐layered materials such as MXene and GO films, nacre, and seashells. For the first time, all five independent elastic properties have been obtained for such multi‐layered materials. The results show clearly that the dimensionless graphene platelet size, the graphene area fraction, and the number of graphene platelet layers can all significantly affect the elastic properties of MGPFs. Other parameters such as graphene platelet regularity and different types of defects could have moderate effects on the elastic properties of MGPFs. The in‐plane elastic properties (i.e., *E*_1_ and *v*_12_) of MGPFs are dominated by those of the graphene platelets when *d*_0_/*l*_0_ is large or by those of the equivalent solid layer material for the van der Waals interaction when *d*_0_/*l*_0_ is small. In contrast, the out‐of‐plane elastic properties (i.e., *E*_3_, *G*_31_ and *v*_31_) of MGPFs are always dominated by those of the equivalent solid layer material for the van der Waals interaction whether *d*_0_/*l*_0_ is large or small. The results in this work help pave the way to achieve the maximum possible elastic properties of these different types of multi‐layered materials.

## Conflict of Interest

The authors declare no competing interests.

## Author Contributions

P.Q. and X.C. contributed equally to this work. X.C., P.Q., Y.L., and B.Z. programmed the computer code; P.Q. and X.C. performed the computational simulations and plotted the figures; H.Z. conceived, designed, and supervised the research; all authors contributed to discussions and analyses of the research work; H.Z., P.Q., and X.C drafted the research results, and H.Z. wrote the paper with input from all authors.

## Supporting information



Supporting Information

## Data Availability

The data that support the findings of this study are available from the corresponding author upon reasonable request.

## References

[1] K. S. Novoselov , A. K. Geim , S. V. Morozov , D. Jiang , Y. Zhang , S. V. Dubonos , I. V. Grigorieva , A. A. Firsov , Science 2004, 306, 666. 15499015 10.1126/science.1102896

[2] C. Lee , X. Wei , J. W. Kysar , J. Hone , Science 2008, 321, 385. 18635798 10.1126/science.1157996

[3] D. G. Papageorgiou , I. A. Kinloch , R. J. Young , Prog. Mater. Sci. 2017, 90, 75.

[4] Y. Lu , G. Yang , S. Wang , Y. Zhang , Y. Jian , L. He , T. Yu , H. Luo , D. Kong , Y. Xianyu , B. Liang , T. Liu , X. Ouyang , J. Yu , X. Hu , H. Yang , Z. Gu , W. Huang , K. Xu , Nat. Electron. 2024, 7, 51.

[5] Y. Zhao , L. Lin , Science 2024, 386, 144. 39388573 10.1126/science.ads4149

[6] G. Yuan , D. Lin , Y. Wang , X. Huang , W. Chen , X. Xie , J. Zong , Q.‐Q. Yuan , H. Zheng , D. Wang , J. Xu , S.‐C. Li , Y. Zhang , J. Sun , X. Xi , L. Gao , Nature 2020, 577, 204. 31915394 10.1038/s41586-019-1870-3

[7] P. Li , M. Yang , Y. Liu , H. Qin , J. Liu , Z. Xu , Y. Liu , F. Meng , J. Lin , F. Wang , C. Gao , Nat. Commun. 2020, 11, 2645. 32461580 10.1038/s41467-020-16494-0 PMC7253461

[8] B. Yuan , Y. Wang , G. Chen , F. Yang , H. Zhang , C. Cao , B. Zuo , J. Hazard. Mater. 2021, 403, 123645. 32853891 10.1016/j.jhazmat.2020.123645

[9] J. Rao , Z. Lv , X. Yan , J. Pan , G. Chen , B. Lü , F. Peng , Adv. Funct. Mater. 2024, 34, 2309869.

[10] C.‐F. Cao , B. Yu , J. Huang , X.‐L. Feng , L.‐Y. Lv , F.‐N. Sun , L.‐C. Tang , J. Feng , P. Song , H. Wang , ACS Nano 2022, 16, 20865. 36468754 10.1021/acsnano.2c08368

[11] G. Xiao , H. Li , Z. Yu , H. Niu , Y. Yao , Nano‐Micro Lett. 2023, 16, 17. 10.1007/s40820-023-01252-w PMC10656391 37975956

[12] Y. Wang , F. Meng , F. Huang , Y. Li , X. Tian , Y. Mei , Z. Zhou , ACS Appl. Mater. Interfaces 2020, 12, 47811. 32985859 10.1021/acsami.0c12501

[13] K. Chen , X. Tang , B. Jia , C. Chao , Y. Wei , J. Hou , L. Dong , X. Deng , T.‐H. Xiao , K. Goda , L. Guo , Nat. Mater. 2022, 21, 1121. 35798946 10.1038/s41563-022-01292-4

[14] M. Wang , M. Huang , D. Luo , Y. Li , M. Choe , W. K. Seong , M. Kim , S. Jin , M. Wang , S. Chatterjee , Y. Kwon , Z. Lee , R. S. Ruoff , Nature 2021, 596, 519. 34433942 10.1038/s41586-021-03753-3

[15] X. Zhang , T. Wu , Q. Jiang , H. Wang , H. Zhu , Z. Chen , R. Jiang , T. Niu , Z. Li , Y. Zhang , Z. Qiu , G. Yu , A. Li , S. Qiao , H. Wang , Q. Yu , X. Xie , Small 2019, 15, 1805395. 10.1002/smll.201805395 30942946

[16] X. Li , W. Cai , J. An , S. Kim , J. Nah , D. Yang , R. Piner , A. Velamakanni , I. Jung , E. Tutuc , S. K. Banerjee , L. Colombo , R. S. Ruoff , Science 2009, 324, 1312. 19423775 10.1126/science.1171245

[17] W. Tian , A. Vahid Mohammadi , Z. Wang , L. Ouyang , M. Beidaghi , M. M. Hamedi , Nat. Commun. 2019, 10, 2558. 31186411 10.1038/s41467-019-10631-0 PMC6560128

[18] W. Li , T. Zhou , Z. Zhang , L. Li , W. Lian , Y. Wang , J. Lu , J. Yan , H. Wang , L. Wei , Q. Cheng , Science 2024, 385, 62. 38963844 10.1126/science.ado4257

[19] H. M. Yang , S. Jo , J. H. Oh , B.‐H. Choi , J. Y. Woo , C.‐S. Han , ACS Nano 2022, 16, 10509. 35820202 10.1021/acsnano.2c01667

[20] L. Cao , C. Wang , Y. Huang , Chem. Eng. J. 2023, 454, 140094.

[21] X. Meng , H. Pan , C. Zhu , Z. Chen , T. Lu , D. Xu , Y. Li , S. Zhu , ACS Appl. Mater. Interfaces 2018, 10, 22611. 29888597 10.1021/acsami.8b05514

[22] Y. Wen , M. Wu , M. Zhang , C. Li , G. Shi , Adv. Mater. 2017, 29, 1702831. 10.1002/adma.201702831 28892207

[23] Y. Chen , H. Qin , H. Liu , L. Shui , Y. Liu , X. Chen , J. Mech. Phys. Solids 2022, 159, 104728.

[24] Y. Chen , H. Liu , K. Pang , C. Zhang , H. Qin , Z. Xu , Y. Liu , J. Mech. Phys. Solids 2023, 171, 105132.

[25] Y. Liu , B. Xie , Z. Zhang , Q. Zheng , Z. Xu , J. Mech. Phys. Solids 2012, 60, 591.

[26] Z. Q. Zhang , B. Liu , Y. Huang , K. C. Hwang , H. Gao , J. Mech. Phys. Solids 2010, 58, 1646.

[27] H. Gao , B. Ji , I. L. Jäger , E. Arzt , P. Fratzl , Proc. Natl. Acad. Sci 2003, 100, 5597. 12732735 10.1073/pnas.0631609100 PMC156246

[28] H. Tang , F. Barthelat , H. D. Espinosa , J. Mech. Phys. Solids 2007, 55, 1410.

[29] H. X. Zhu , S. M. Thorpe , A. H. Windle , Philos. Mag. A 2001, 81, 2765.

[30] H. X. Zhu , J. R. Hobdell , A. H. Windle , J. Mech. Phys. Solids 2001, 49, 857.

[31] J. Zhan , Z. Lei , Y. Zhang , Chem 2022, 8, 947.

[32] M. Yang , Y. Liu , T. Fan , D. Zhang , Prog. Mater. Sci. 2020, 110, 100652.

[33] A. Crisafulli , A. Khodayari , S. Mohammadnejad , M. Fasano , Crystals 2018, 8, 149.

[34] Z. Xue , G. Chen , C. Wang , R. Huang , J. Mech. Phys. Solids 2022, 158, 104698.

[35] H. Yang , B. Martín‐García , J. Kimák , E. Schmoranzerová , E. Dolan , Z. Chi , M. Gobbi , P. Němec , L. E. Hueso , F. Casanova , Nat. Mater. 2024, 23, 1502. 39191981 10.1038/s41563-024-01985-y

[36] X. Liu , Z. Bie , P. Yu , B. Zheng , X. Shi , Y. Fan , X. He , C. Lu , Compos. Struct. 2024, 332, 117926.

[37] X. Zhang , W. Lu , G. Zhou , Q. Li , Adv. Mater. 2020, 32, 1902028. 10.1002/adma.201902028 31250496

[38] S. Wan , L. Jiang , Q. Cheng , Matter 2020, 3, 696.

[39] L. Ruiz , W. Xia , Z. Meng , S. Keten , Carbon 2015, 82, 103.

[40] S. Wang , Y. Chen , Y. Ma , Z. Wang , J. Zhang , J. Appl. Phys. 2017, 122, 074301.

[41] A. Dey , A. Azizimanesh , S. M. Wu , H. Askari , ACS Appl. Mater. Interfaces 2024, 16, 8169. 38295436 10.1021/acsami.3c19101 PMC10875650

[42] J. H. Lee , A. Avsar , J. Jung , J. Y. Tan , K. Watanabe , T. Taniguchi , S. Natarajan , G. Eda , S. Adam , A. H. Castro Neto , B. Özyilmaz , Nano Lett. 2015, 15, 319. 25493357 10.1021/nl5036012

[43] E. Koren , E. Lörtscher , C. Rawlings , A. W. Knoll , U. Duerig , Science 2015, 348, 679. 25954007 10.1126/science.aaa4157

[44] Y. Wei , B. Wang , J. Wu , R. Yang , M. L. Dunn , Nano Lett. 2013, 13, 26. 23214980 10.1021/nl303168w

[45] Y. Shen , H. Wu , Appl. Phys. Lett. 2012, 100, 101909.

[46] P. H. Tan , W. P. Han , W. J. Zhao , Z. H. Wu , K. Chang , H. Wang , Y. F. Wang , N. Bonini , N. Marzari , N. Pugno , G. Savini , A. Lombardo , A. C. Ferrari , Nat. Mater. 2012, 11, 294. 22306771 10.1038/nmat3245

[47] E. Han , J. Yu , E. Annevelink , J. Son , D. A. Kang , K. Watanabe , T. Taniguchi , E. Ertekin , P. Y. Huang , A. M. Zande , Nat. Mater. 2020, 19, 305. 31712745 10.1038/s41563-019-0529-7

[48] Q. Peng , C. Liang , W. Ji , S. De , Phys. Chem. Chem. Phys. 2013, 15, 2003. 23257777 10.1039/c2cp43360e

[49] G. Wang , Z. Dai , J. Xiao , S. Feng , C. Weng , L. Liu , Z. Xu , R. Huang , Z. Zhang , Phys. Rev. Lett. 2019, 123, 116101. 31573244 10.1103/PhysRevLett.123.116101

[50] P. H. Tan , W. P. Han , W. J. Zhao , Z. H. Wu , K. Chang , H. Wang , Y. F. Wang , N. Bonini , N. Marzari , N. Pugno , G. Savini , A. Lombardo , A. C. Ferrari , Nat. Mater. 2012, 11, 294. 22306771 10.1038/nmat3245

[51] H. X. Zhu , C. Y. Chen , Mech. Mater. 2011, 43, 276.

[52] M. Mohi Uddin , M. Humaun Kabir , M. Ashraf Ali , M. Mukter Hossain , M. Uddin Khandaker , S. Mandal , A. Arifutzzaman , D. Jana , RSC Adv. 2023, 13, 33336. 37964903 10.1039/d3ra04456d PMC10641765

[53] Z. Ding , T. Klein , C. Barner‐Kowollik , M. Mirkhalaf , Mater. Horiz. 2023, 10, 5371. 37882614 10.1039/d3mh01015e

[54] Z. He , Y. Zhu , J. Xia , H. Wu , J. Mech. Phys. Solids 2019, 133, 103706.

[55] W. Xia , L. Ruiz , N. M. Pugno , S. Keten , Nanoscale 2016, 8, 6456. 26935048 10.1039/c5nr08488a

[56] N. Liu , J. Hong , X. Zeng , R. Pidaparti , X. Wang , Phys. Chem. Chem. Phys. 2017, 19, 13083. 28484774 10.1039/c7cp01033h

[57] H. J. Lei , Z. Q. Zhang , F. Han , B. Liu , Y.‐W. Zhang , H. J. Gao , J. Appl. Mech. 2013, 80, 061017.

[58] S. Anup , J. Mech. Behav. Biomed. Mater. 2015, 46, 168. 25792414 10.1016/j.jmbbm.2015.02.026

[59] M. Maghsoudi‐Ganjeh , L. Lin , X. Yang , X. Zeng , J. Mater. Res. 2021, 36, 2651.

[60] F. Barthelat , J. Mech. Phys. Solids 2014, 73, 22.

[61] F. Barthelat , H. Tang , P. D. Zavattieri , C.‐M. Li , H. D. Espinosa , J. Mech. Phys. Solids 2007, 55, 306.

[62] R. Menig , M. H. Meyers , M. A. Meyers , K. S. Vecchio , Acta Mater. 2000, 48, 2383.

[63] D. Jiao , Z. Q. Liu , Y. K. Zhu , Z. Y. Weng , Z. F. Zhang , Mater. Sci. Eng.: C 2016, 68, 9. 10.1016/j.msec.2016.05.089 27523990

[64] F. Barthelat , C.‐M. Li , C. Comi , H. D. Espinosa , J. Mater. Res. 2006, 21, 1977.

[65] F. Song , J. Zhou , X. Xu , Y. Xu , Y. Bai , Phys. Rev. Lett. 2008, 100, 245502. 18643597 10.1103/PhysRevLett.100.245502

[66] J. Wang , Q. Cheng , L. Lin , L. Jiang , ACS Nano 2014, 8, 2739. 24506706 10.1021/nn406428n

