# Supplementary material for: Quantifying the Effects of Geometric Parameters on the Elastic Properties of Multilayer Graphene Platelet Films
Source: Adv Mater. 2025 Jun 2;37(33):2502546. doi: 10.1002/adma.202502546 (PMC12369695; doi:10.1002/adma.202502546)
Supplement: Supplementary file 1 — Supporting Information [file ADMA-37-2502546-s001.docx]

Supporting Information

Quantifying the effects of geometric parameters on the elastic properties of multilayer graphene platelet films

Penghao Qi, Xindong Chen, Hanxing Zhu, Yongtao Lyu, Bu Zhang, Qing Peng, Xiqiao Feng, Tongxiang Fan, Di Zhang

This supplementary material provides some additional information about the relevant parameters of the geometric models, the finite element simulation setups as well as some simulation data and results, which could validate the reasonableness of the geometric models, and demonstrate the dominant deformation mechanisms of the MGPFs.

**The PDF file includes:**

Supplementary notes

Figures S1 to S6

Tables S1 to S5

References

The single layer graphene platelet structure in 3D RVE model of MGPFs

**Figure S1** shows the geometric structure in a single layer of the 3D representative volume element (RVE) model (as shown in Figure 1c) of multilayer graphene platelet films (MGPFs), which is a periodic 2D square random irregular Voronoi graphene platelet model with a specific degree of graphene platelet regularity defined in Eq. (2). For simplicity, all the boundaries between the intralayer graphene platelets are assumed to have the same uniform gap as shown by the solid lines in Figure S1, thus it is easy to control the graphene area fraction $F_{A}$ defined in Eq. (3) by specifying the gap width of the intralayer graphene platelet boundaries.


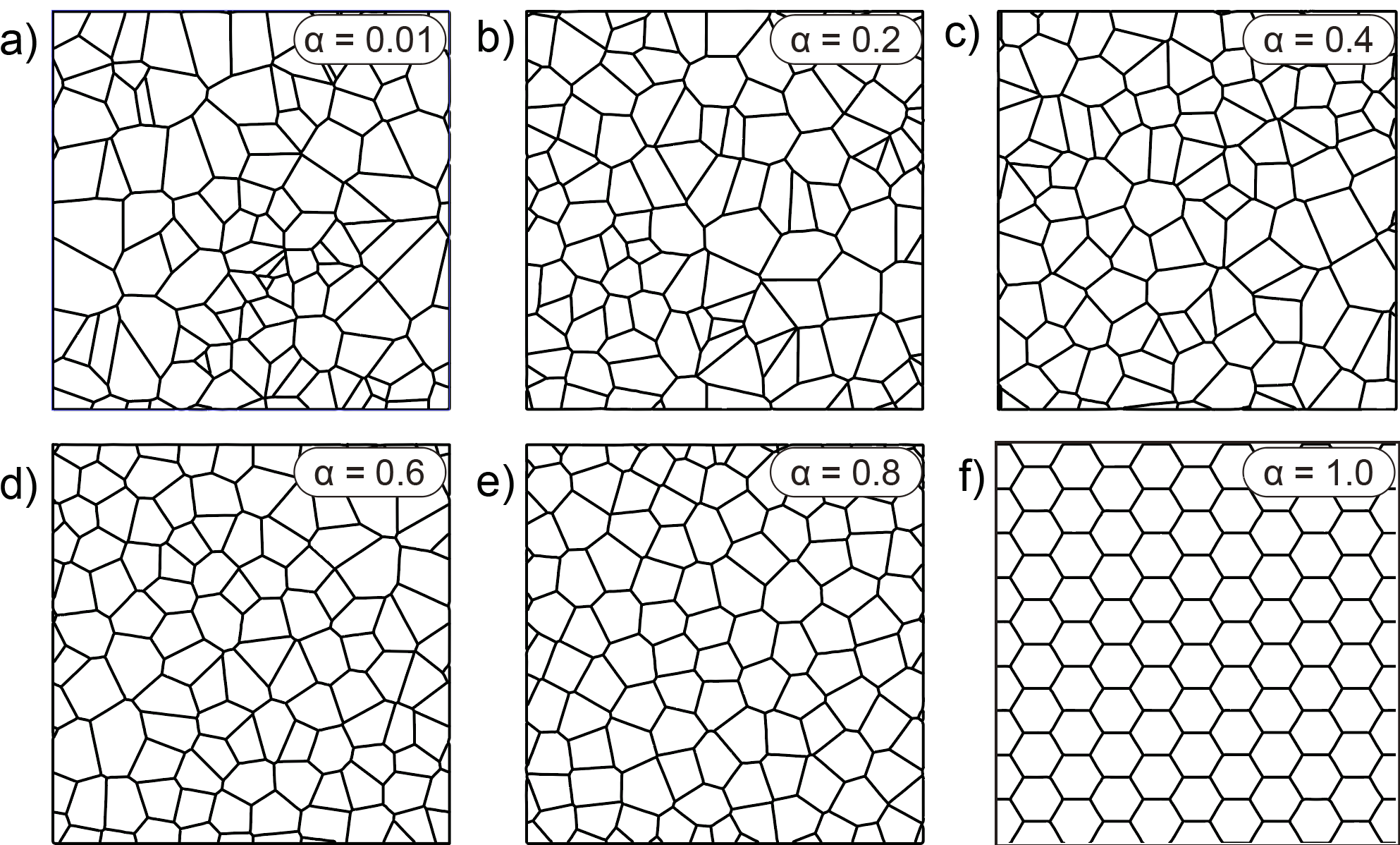


**Figure S1.** Periodic 2D square random irregular Voronoi graphene platelet model with 100 complete graphene platelets and different degrees of regularity α. (a) α=0.01, (b) α=0.2, (c) α=0.4, (d) α=0.6, (e) α=0.8 and (f) α=1.0.

**Equivalent parameters for shell elements of graphene**

The elastic properties of monolayer graphene are in general considered in-plane isotropic. To accurately describe the mechanical behavior of monolayer graphene using the classical ABAQUS S4R shell elements, it is essential to ensure the bending stiffness, the in-plane tensile stiffness and Poisson’s ratio of the S4R shell elements to be the same as those of the monolayer graphene. The bending stiffness is typically between 1.2 to 1.7 eV^[1,2]^ (i.e., B=$(1.92\sim2.72)\times10^{-19}$ Nm) for monolayer graphene, therefore, the in-plane tensile stiffness $D=340N/m$^[3]^, Poisson’s ratio $v=0.178$ and the bending stiffness $B=2.385\times10^{-19}$Nm are adopted . Assuming the Young’s modulus of the shell solid and the thickness of the shell elements are $E_{S}$ and $t$, the relations $E_{S}t=D$ and $B=E_{S}t^{3}/[12(1-v^{2})]$ must be satisfied to ensure the same stretching and bending stiffnesses of monolayer graphene. Thus, the equivalent Young’s modulus of the shell solid material is obtained as $E_{S}=3765.6$ GPa, and the equivalent thickness of S4R shell elements is determined as $t=\sqrt{12B(1-v^{2})/D}=0.09029$ nm.

**Finite element setup**

All simulations were performed using the *Static, General* module in Abaqus under linear elastic conditions. The loading was applied in the form of displacement-induced strain, with the strain values kept at 0.001 to ensure accurate extraction of linear elastic properties.

The RVE models were constructed using the Voronoi tessellation method proposed by Zhu et al.^[4]^, which enables the generation of in-plane periodic Voronoi structures with controllable geometric parameters such as degree of regularity. In the finite element method, to ensure that the boundary nodes are perfectly matched for applying periodic boundary conditions, the graphene platelets were meshed using the *Advanced Front* algorithm. Boundary nodes were identified through a Python script, and corresponding node pairs were matched based on a coordinate mapping tolerance of less than 0.00001. Subsequently, following and modifying the modelling approach described in the literature^[5]^, periodic boundary conditions were implemented in the *Interaction* module of Abaqus using the *Equation* method. Details are provided as follows:

Under different loading conditions, reference points RP-1 and RP-2 were defined in the model plane, as shown in Figure S2b, to control the in-plane boundary conditions in the x and y directions for the boundary nodes of each graphene layer. Taking the 2D periodic structure in Figure S2b as an example, the boundary nodes on the corresponding edges of the RVE are paired, meaning that periodic boundary conditions are applied on the four edges of the RVE, namely $\Gamma_{l},\Gamma_{r},\Gamma_{b}$ and $\Gamma_{t}$. Assuming that two paired nodes *i* (Node-Left on $\Gamma_{l}$) and *j* (Node-Right on $\Gamma_{r}$) have displacements $u_{i}, v_{i}, u_{j}$ and $v_{j}$ and that a uniaxial strain is applied in the horizontal (x) direction while the other directions remain unstrained, the periodic boundary conditions on $\Gamma_{l}$ and $\Gamma_{r}$ can be written as

|  | $u_{i}-u_{j}=u_{Ref1}$ |  |
| --- | --- | --- |
|  | $v_{i}-v_{j}=v_{Ref1}$ |  |

Similarly, the nodal displacements in the Y direction follow the corresponding relationships.

|  | $u_{k}-u_{l}=u_{Ref2}$ |  |
| --- | --- | --- |
|  | $v_{k}-v_{l}=v_{Ref2}$ |  |

Where, $u_{Ref1}$, $v_{Ref1}$*,* $u_{Ref2}$ and $v_{Ref2}$ represent the x and y displacements of the two reference points Ref1 and Ref2. When the displacement of a reference point is set to zero, it indicates that the structure does not expand or contract in that direction. However, in this study, such a constraint would lead to over-constraining the system. To account for the Poisson effect, no displacement constraints are imposed on the reference points in this direction. To obtain the in-plane Young’s modulus and Poisson’s ratio, one node on the bottom left edge is fixed to prevent rigid translation of the RVE, and a displacement of $u_{Ref1}=0.001L$ in the x direction is applied, where *L* is the in-plane side length of the RVE as shown in Figure S2b below. According to ABAQUS simulation results of the corresponding nodal force in the x direction and the corresponding $v_{Ref2}$ value, the in-plane Young’s modulus and Poisson’s ratio of the MGPF can be calculated.


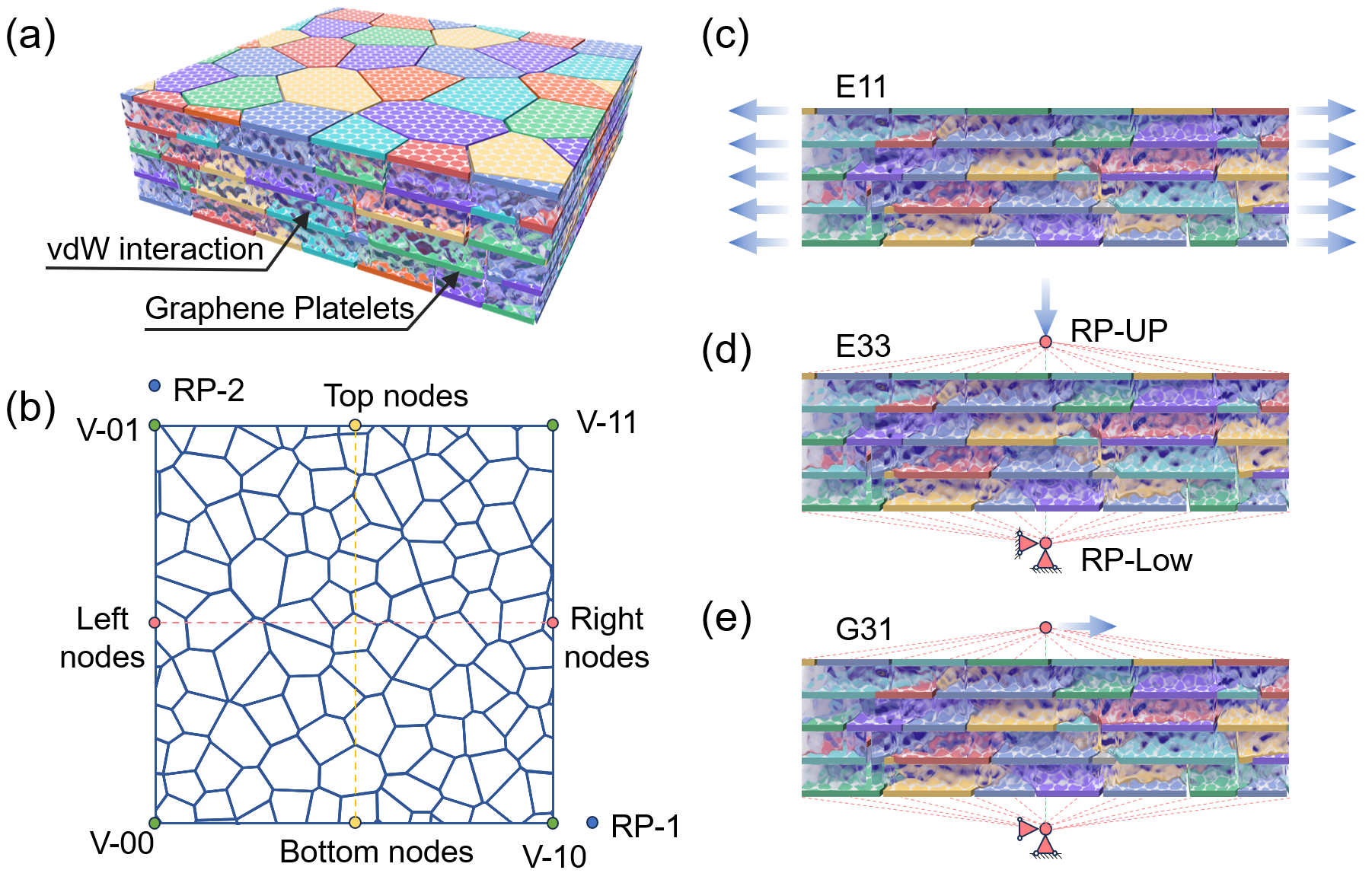


**Figure S2** Examples of node set categorisation conditions for 2D periodic structure. (a) Schematic of MGPFs model, (b) PBC setup, and (c) in-plane tension (d) out-of-plane compression, (e) out-of-plane shear loading.

**Loading types:**

Three primary loading conditions are considered in the model: in-plane tension (to obtain $E_{1}$ and $v_{12}$), out-of-plane compression (to obtain $E_{3}$ and $v_{1}$ ), and out-of-plane shear (to obtain $G_{31}$). The loading method is illustrated in Figures S2c, d and e. It should be noted that the model is loaded by applying displacement, and the corresponding stress response is calculated from the extracted reaction forces.

Determination of the size of RVE models

The in-plane properties of graphene platelet sheets are typically isotropic, while simulation results may be influenced by the number *N* of complete graphene platelets per layer in the RVE model, which is a model-specific parameter rather than an intrinsic material property. The number *N* of complete Voronoi graphene platelets in each layer of the RVE models should be sufficiently large to ensure the in-plane elastic properties of MGPFs obtained from the simulations to be isotropic. However, the larger the number *N*, the more expensive the computational simulations. For RVE models with an area fraction $F_{A}$ close to 1.0, an in-plane side length *L* and different regularity degrees of the Voronoi graphene platelets, the mean diameter of the graphene platelets is defined as *d_0_* and given by Eq. (1). In order to decide a suitable number *N*, finite element simulations are performed to obtain the in-plane Young’s module and Poisson’s ratios for RVE models with different numbers of *N*, and the results are presented in **Table S1**. It is noted that each of the data in Table S1 is the mean result obtained from 20 similar random RVE models with the regularity degree of the Voronoi graphene platelets fixed at ***α***=0.6, the number of the graphene platelet layers fixed at $M=5$, the mean diameter of the graphene platelets fixed at *d_0_*=106.5 nm, and the uniform gap between the intralayer neighboring graphene platelets fixed at 1 nm. Thus, the larger the number *N* of the graphene platelets in each layer, the larger the in-plane side length *L* of the RVE models in the simulations of Table S1. As can be seen from Table S1, the effects of number *N* of the complete graphene platelets in each layer on the in-plane elastic properties of the MGPFs are not significant. Thus, the number *N* of the complete graphene platelets in each layer of the REV models is fixed at 100 for all the finite element simulations in this work. It is worth noting that it is impractical to use atomistic simulations to study the effects of the different geometric parameters on the elastic properties of MGPFs as most of the individual finite element RVE models contain over one billion atoms and we need to perform thousands of simulations to obtain all the relevant results in this work.

**Table S1**. Effects of the number of complete graphene platelets in each layer of the RVE models on the in-plane elastic properties of MGPFs with five graphene layers.

| Number of graphene plates $N$ | Mean $E_{1}$ (GPa) | Std. dev. | Mean $\nu_{12}$ | Std. dev. |
| --- | --- | --- | --- | --- |
| 36 | 894.8055511 | 1.472380621 | 0.166046238 | 0.000350819 |
| 64 | 895.8474373 | 0.477627034 | 0.166265245 | 0.000324648 |
| 100 | 895.6296611 | 0.714900395 | 0.166336464 | 0.000138028 |
| 225 | 895.6952465 | 0.451358181 | 0.166610428 | 0.000168993 |
| 400 | 895.8426718 | 0.298868222 | 0.166609422 | 0.000280676 |

The number of independent elastic properties of MGPFs

In this research work, each data point is obtained from 20 similar 3D and in-plane periodic random RVE models with the same combination of the different geometric parameters, as illustrated in **Tables S2** and **S3**. In Table S2, the values of the in-plane elastic properties of MGPFs, $E_{1}$, $G_{12}$ and $v_{12}$, are obtained by finite element simulations. As can been seen, these three in-plane elastic properties obviously satisfy the relation $E_{1}=2G_{12}(1+v_{12})$, thus the elastic properties of MGPFs are in-plane isotropic and only two of the three in-plane elastic constants are independent. As MGPFs have three orthogonal planes of elastic symmetry and one of these planes is elastic isotropic, the MGPFs have only five independent elastic constants (properties) to be determined in this work. These are $E_{1}$, $v_{12}$, $E_{3}$, $G_{31}$ and $v_{31}$ as shown in Table S3. For the first time, we obtain all these five independent elastic properties and quantify the effects of the different geometric parameters on the elastic properties of MGPFs. This work could help pave the way to achieve the best or desired elastic properties of MGPFs.

**Table S2.** The isotropic in-plane elastic properties obtained from 20 similar random periodic RVE models of MGPFs with a mean graphene platelet diameter *d*_0_= 106.5 nm (i.e. *d*_0_/ $l_{0}$ = 44.64), a regularity degree ***α***=0.6 and the number of graphene platelet layers *M*=5.

| Model NO. | $E_{1}$ (GPa) | $\nu_{12}$ | $E_{1}$/$[2\left( 1+v_{12} \right)]$ (GPa) | $G_{12}$ (GPa) |
| --- | --- | --- | --- | --- |
| 1 | 895.360 | 0.166187 | 383.884 | 383.759 |
| 2 | 895.321 | 0.166286 | 383.834 | 383.548 |
| 3 | 895.702 | 0.166182 | 384.032 | 383.512 |
| 4 | 896.933 | 0.166269 | 384.531 | 384.086 |
| 5 | 895.522 | 0.166126 | 383.973 | 384.039 |
| 6 | 895.551 | 0.166259 | 383.942 | 384.182 |
| 7 | 894.580 | 0.166255 | 383.527 | 384.072 |
| 8 | 896.686 | 0.166661 | 384.296 | 384.496 |
| 9 | 896.737 | 0.166326 | 384.428 | 383.957 |
| 10 | 895.216 | 0.166237 | 383.806 | 383.745 |
| 11 | 894.721 | 0.166250 | 383.589 | 383.900 |
| 12 | 896.299 | 0.166422 | 384.209 | 384.625 |
| 13 | 894.888 | 0.166298 | 383.645 | 383.995 |
| 14 | 895.310 | 0.166311 | 383.821 | 384.083 |
| 15 | 896.173 | 0.166382 | 384.168 | 383.684 |
| 16 | 894.897 | 0.166531 | 383.572 | 383.508 |
| 17 | 894.951 | 0.166411 | 383.635 | 383.911 |
| 18 | 895.299 | 0.166437 | 383.775 | 383.862 |
| 19 | 896.517 | 0.166314 | 384.338 | 384.536 |
| 20 | 895.930 | 0.166587 | 383.996 | 384.225 |
| Mean | 895.630 | 0.166336 | 383.950 | 383.986 |
| Std. dev. | 0.69680 | 0.000135 | 0.34835 | 0.31387 |

**Table S3.** The five independent elastic constants obtained from 20 similar random periodic RVE models of MGPFs with a mean graphene platelet diameter *d*_0_= 106.5 nm (i.e. *d*_0_/ $l_{0}$ = 44.64), a regularity degree ***α***=0.6 and the number of graphene platelet layers *M*=5.

| Model NO. | $E_{1}$ (GPa) | $\nu_{12}$ | $E_{3}$ (GPa) | $\nu_{31}$ | $G_{31}$ (MPa) |
| --- | --- | --- | --- | --- | --- |
| 1 | 895.360 | 0.166187 | 9.438893 | 0.000721 | 4771.072 |
| 2 | 895.321 | 0.166286 | 9.443134 | 0.000836 | 4773.267 |
| 3 | 895.702 | 0.166182 | 9.432347 | 0.000016 | 4768.457 |
| 4 | 896.933 | 0.166269 | 9.438139 | 0.000016 | 4771.019 |
| 5 | 895.522 | 0.166126 | 9.441792 | 0.000039 | 4773.631 |
| 6 | 895.551 | 0.166259 | 9.438368 | 0.000125 | 4770.771 |
| 7 | 894.580 | 0.166255 | 9.441900 | 0.000587 | 4772.645 |
| 8 | 896.686 | 0.166661 | 9.433628 | 0.000064 | 4768.757 |
| 9 | 896.737 | 0.166326 | 9.431642 | 0.000227 | 4768.323 |
| 10 | 895.216 | 0.166237 | 9.440695 | 0.000102 | 4777.114 |
| 11 | 894.721 | 0.166250 | 9.432639 | 0.000027 | 4770.672 |
| 12 | 896.299 | 0.166422 | 9.431003 | 0.000101 | 4770.317 |
| 13 | 894.888 | 0.166298 | 9.442563 | 0.000261 | 4773.229 |
| 14 | 895.310 | 0.166311 | 9.431594 | 0.000131 | 4777.209 |
| 15 | 896.173 | 0.166382 | 9.449629 | 0.000021 | 4773.564 |
| 16 | 894.897 | 0.166531 | 9.443346 | 0.000406 | 4770.113 |
| 17 | 894.951 | 0.166411 | 9.442952 | 0.000214 | 4772.319 |
| 18 | 895.299 | 0.166437 | 9.441388 | 0.000136 | 4773.220 |
| 19 | 896.517 | 0.166314 | 9.439814 | 0.000044 | 4772.620 |
| 20 | 895.930 | 0.166587 | 9.436955 | 0.000088 | 4775.340 |
| Mean | 895.630 | 0.166336 | 9.438621 | 0.000208 | 4772.183 |
| Std. dev. | 0.69680 | 0.000135 | 0.004958 | 0.000236 | 2.474555 |

Construction of RVE models with different graphene area fractions

Graphene area fraction $F_{A}$ defined in Equation (3) is an important geometric parameter which could significantly affect the elastic properties of MGPFs. In order to more accurately quantify its effects, it is critical to fix other geometric parameters and minimize their possible effects on the elastic properties of MGPFs. For simplicity, all the boundaries between the intralayer graphene platelets in the RVE model are assumed to have approximately the same uniform gap, thus it is easy to control the graphene area fraction $F_{A}$ according to Equation (3) by specifying the gap width of the intralayer graphene platelet boundaries. The results in **Figure 2** have already shown that when the uniform gap between the intralayer neighboring graphene platelets is fixed at 1nm and if the mean graphene diameter is *d*_0_=500nm, the stiffnesses ($E_{3}$ and $G_{31}$ and $E_{1}$) of MGPFs can reach about 96% - 99.3% of their maximum possible values. To minimize the possibly additional effects of the mean graphene platelet size, its value is thus always fixed at *d*_0_=500 nm in this part of study.

For a RVE model with $d_{0}=500$ nm and $N=100$, if the gap between the intralayer graphene platelets is zero (i.e. $F_{A}=1.0$), the in-plane side length of the RVE model can be determined as $L=4.653 \mu m$ from Equation (1). Random RVE models of MGPFs with an in-plane side length $L=4.653 \mu m$, number of graphene layers $M=5$, regularity degree $\alpha=0.6$ and graphene area fraction $F_{A}=1.0$ are constructed first. For a required value of the graphene platelet area fraction $F_{A}$, the corresponding in-plane side length of the RVE models can be determined as $L=\sqrt{\sqrt{3}N}d_{0}/\sqrt{2F_{A}}$ where $d_{0}=500$ nm and the graphene area fraction $F_{A}\leq1.0$, and the corresponding RVE models can be obtained by shifting the centres of the individual Voronoi polygons (i.e. the centres of the individual graphene platelets) to their right positions according to their original x and y coordinates. For example, when the uniform gap between the intralayer graphene platelets is 1nm and the corresponding graphene platelet area fraction can be obtained as $F_{A}=99.6\%$, the in-plane side length of the RVE model can be determined as $L=4.662 \mu m$. It is noted that the graphene overlap ratio $\rho$ is the total overlap area between any two staggered graphene platelet layers in an RVE model divided by $L^{2}$. Obviously, the overlap area of any two staggered graphene platelets is always smaller than each of these two graphene platelets and *ρ* is always smaller than $F_{A}$ for any RVE model of MGPFs. In addition, *ρ* and $F_{A}$ are two closely corelated geometric parameters, and the smaller the graphene area fraction $F_{A}$, the smaller the graphene overlap ratio *ρ* as can be seen in Figure 3a.

The power law relations between the out-of-plane stiffnesses and $\boldsymbol{F}_{\boldsymbol{A}}$

The out-of-plane stiffnesses $E_{3}$ and $G_{31}$ of MGPFs can well be described by a power function of the graphene area fraction $F_{A}$, e.g. $E_{3}\propto F_{A}^{k}\mathrm{or}G_{31}\propto\rho^{2.5}\propto F_{A}^{5}$. For MGPFs with $d_{0}=500nm$, $\alpha=0.6$ and different numbers of graphene platelet layers *M*, the powers *k* can be determined from their log–log plots according to the relevant results as shown in Figures 3d and 3f, and are presented in **Table S4**, which indicates that the larger the number *M* of the graphene platelet layers, the larger the value of *k* in the power function of $F_{A}^{k}.$

**Table S4.** The power *k* in the power functions of $F_{A}$ for the out-of-plane stiffnesses of MGPFs with $d_{0}=500nm$, $\alpha=0.6$, and different numbers of the graphene platelet layers.

| Model | Number of graphene platelet layers | | |
| --- | --- | --- | --- |
|  | 4 Layers | 5 Layers | 6 Layers |
| $E_{3}$ (GPa) | 3.935 | 5.191 | 6.007 |
| $G_{31}$ (MPa) | 3.901 | 5.065 | 5.803 |

Effects of $\boldsymbol{F}_{\boldsymbol{A}}$ on the von Mises stress contour of the graphene platelets

**Figure S3** presents the comparison between the contour diagrams of the Von Mises stress in graphene platelets of the RVE models with different area fractions under an in-plane uniaxial tensile strain of 0.001, from left to right: F_A_= 0.996, 0.769, 0.591. The RVE models of MGPFs have a mean graphene diameter *d*_0_=500 nm, number of graphene layers *M*=5 and graphene platelet regularity *α*=0.6. As can be seen, the smaller the graphene area fraction $F_{A}$ , the larger the magnitude of the von Mises stress and the more uneven of the stress distribution in different graphene platelets. Stress concentration occrs at the edges of the graphene platelets.


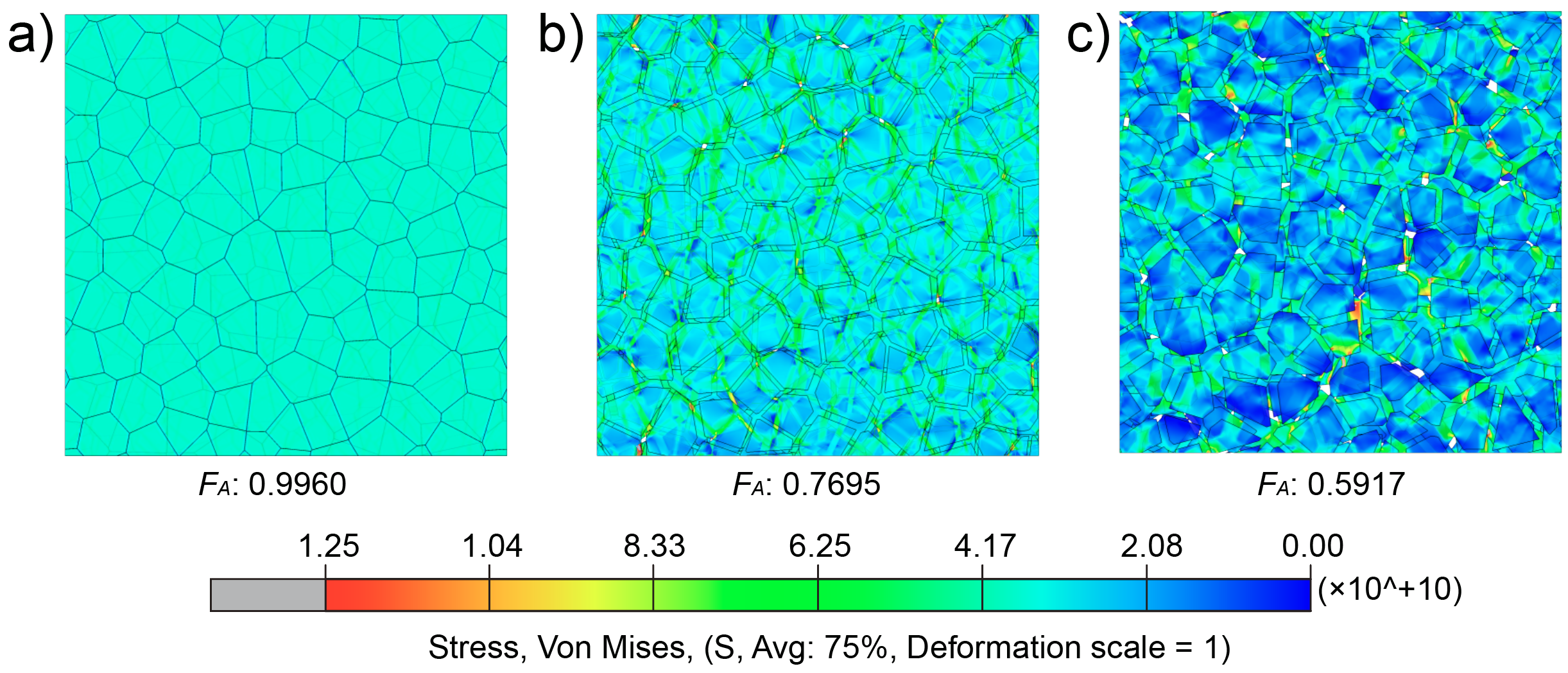


**Figure S3**. Comparison between the contour diagrams of the von Mises stress in graphene platelets of the RVE models with different area fractions, a) $F_{A}=$ 0.9960, b) $F_{A}=$ 0.7695, c) $F_{A}=$ 0.5917, under an in-plane uniaxial tensile strain of 0.001. All the RVE models have the same average graphene platelet diameter *d*_0_=500 nm, number of graphene layers *M*=5 and graphene platelet regularity *α*=0.6.

Effects of $\boldsymbol{F}_{\boldsymbol{A}}$ on the rotational magnitudes of graphene platelets

The RVE models of MGPFs shown in **Figure S4** have a mean graphene diameter *d*_0_=500 nm, 100 complete graphene platelets in each layer, number of graphene layers *M*=5, graphene platelet regularity *α* = 0.6, and different values of graphene area fraction $F_{A}$ = 0.996, 0.769, or 0.591. When the RVE models are under either an out-of-plane shear (Figure S4a) or compressive (Figure S4b) strain, or an in-plane tensile strain (Figure S4c), the graphene platelets in the middle layer of the RVE models could undergo rotational deformation. The contour results in Figure S4 demonstrate that the smaller the graphene area fraction $F_{A}$, the larger the rotational magnitude of the graphene platelets in the middle layer of the RVE model. Therefore, the smaller the graphene area fraction $F_{A}$, the smaller the in-plane Young’s modulus and the out-of-plane Young’s and shear moduli of the MGPFs, further interpreting the results in Figures 3f, 3d and 3b in the main text.


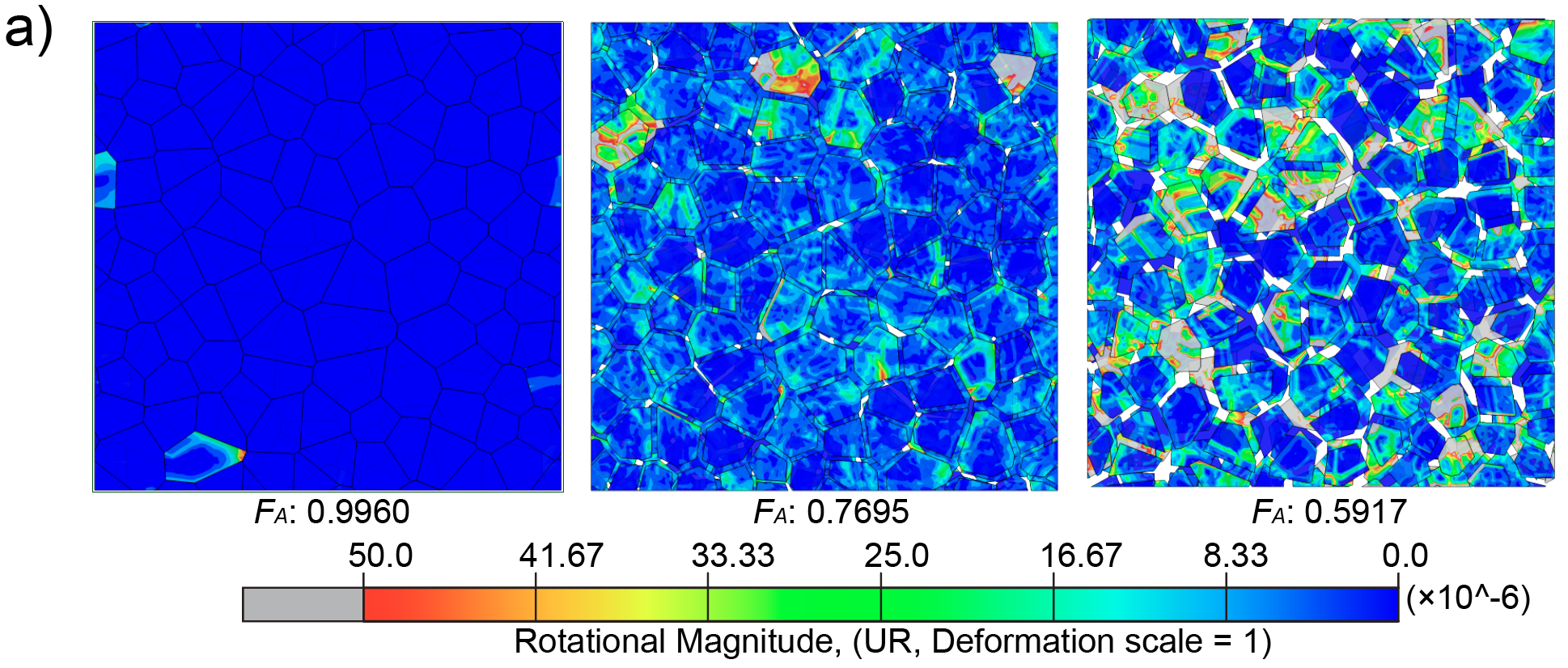


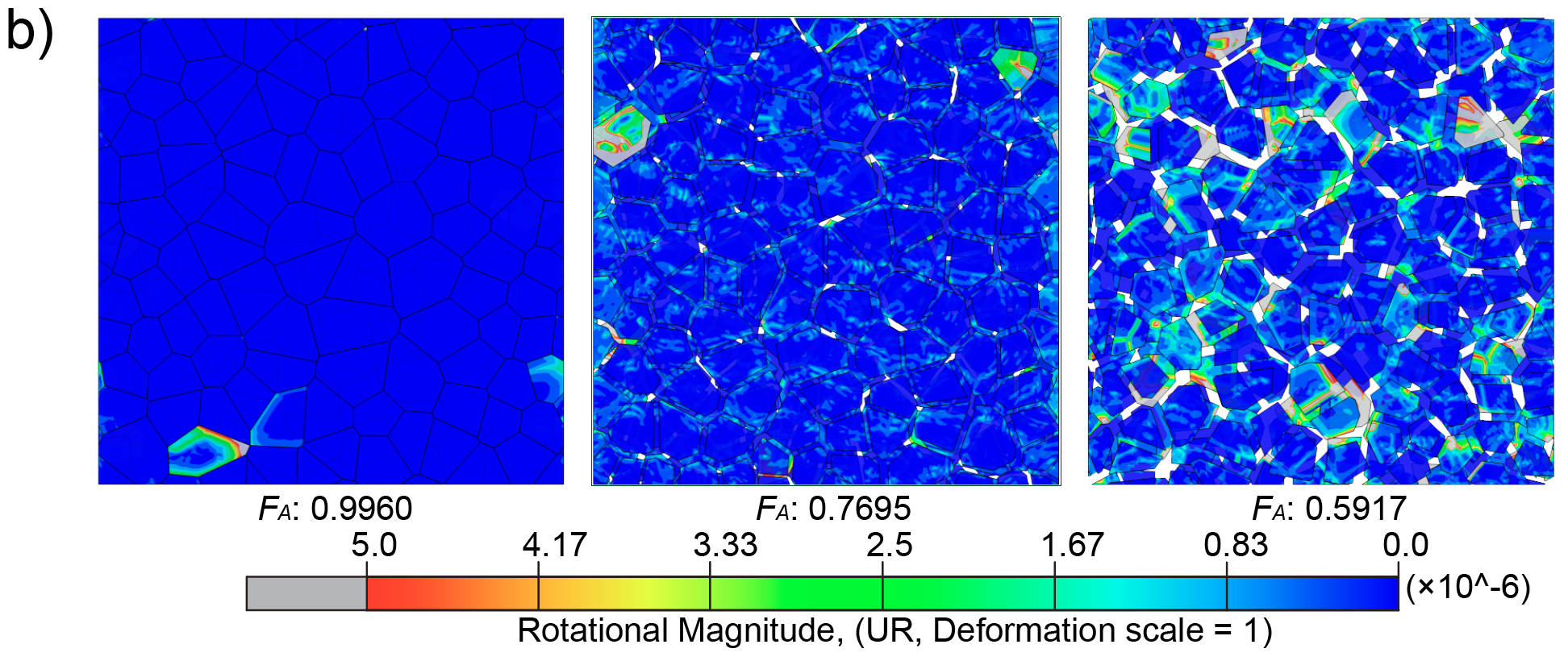


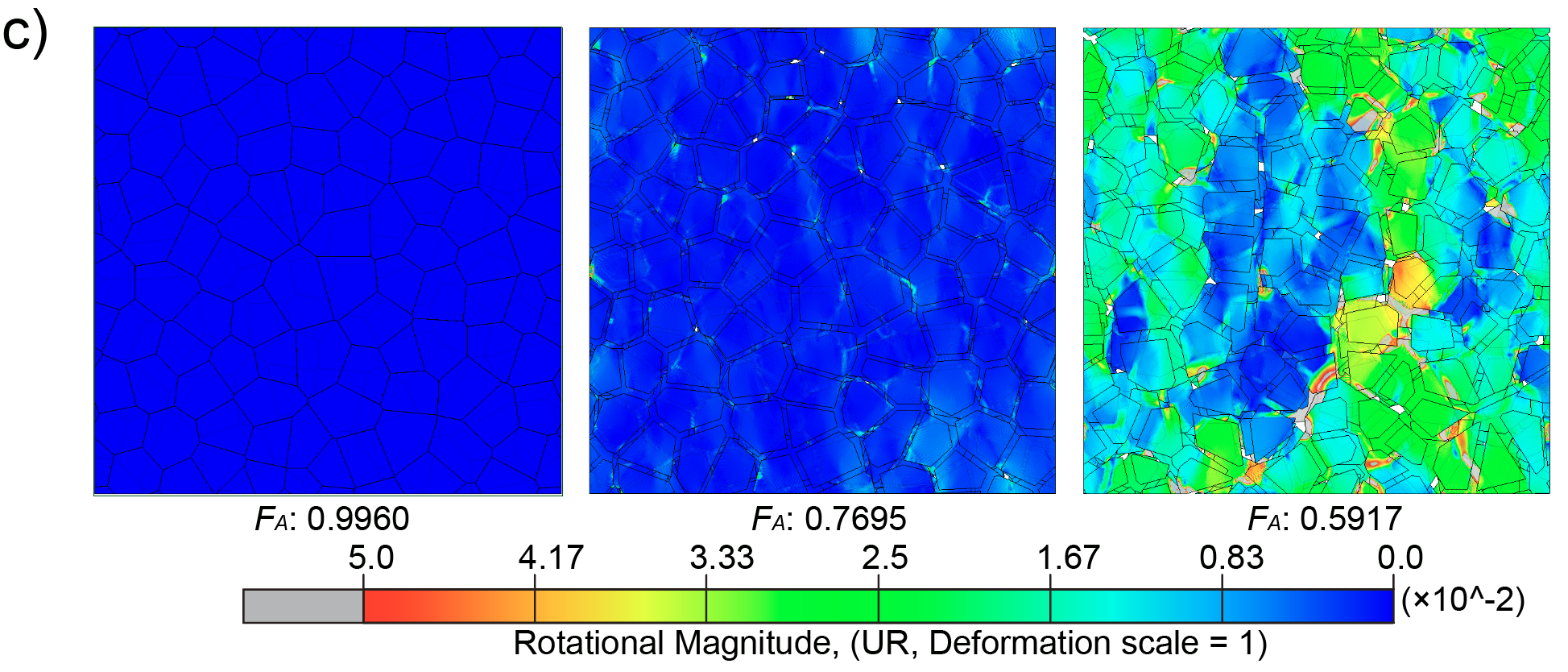


**Figure S4.** Comparison between the contour diagrams of the rotational magnitudes of graphene platelets in the middle layer of the RVE models with the average graphene platelet diameter *d*_0_=500 nm, 100 complete graphene platelets in each layer, number of graphene layers *M*=5, graphene platelet regularity *α*=0.6, and different values of graphene area fraction $F_{A}$ . From left to right: $F_{A}$ =0.996, 0.769, 0.591; from top to bottom, a) under an out-of-plane shear strain of 0.001,  b) under an out-of-plane compressive strain of 0.001, c) under in-plane tensile strain of 0.001.

Effects of the $\mathbf{n}\mathbf{umber}$ of graphene layers *M* on the rotational magnitude of graphene platelets

For RVE models of MGPFs with a mean graphene diameter *d*_0_=500 nm, 100 complete graphene platelets in each layer, graphene platelet regularity *α*=0.6, graphene area fraction $F_{A}=0.591$ and different numbers of graphene platelet layers *M =* 5 or 9, **Figure S5** shows the comparison between the rotational magnitude contours of the graphene platelets in the middle layer of the RVE models when the RVEs are deformed by the same magnitude of out-of-plane compressive or shear stress of 0.1 MPa. The results llustrate clearly that under the same magnitude of out-of-plane conpressive or shear stress (0.1 MPa), the graphene platelets in RVE model with M=9 have a large magnitude of rotation (or are more easily to rotate) than those in RVE model with M=5. Thus, the larger the number of the graphene platelet layers, the smaller the out-of-plane Young’s and shear moduli of the MGPFs, confirming the relevant results in Figure 4.


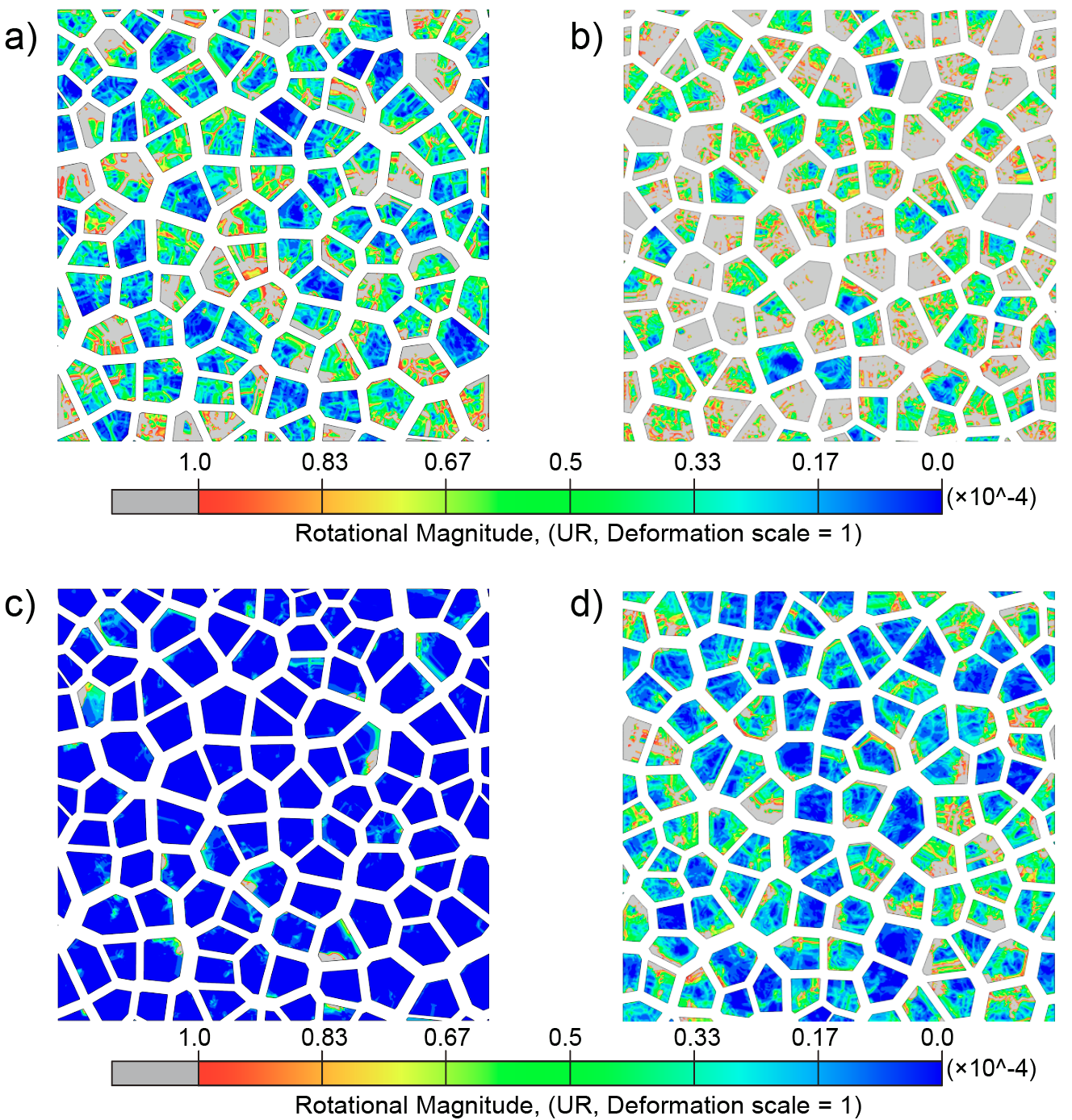


**Figure S5**. Comparison between contour diagrams of the rotational magnitudes of graphene platelets in the middle layer of RVE models with $F_{A}=0.591$ and different numbers of graphene layers *M* under the same out-of-plane compressive or shear stress of 0.1 MPa. Contour diagrams of models with a) *M*=5 and b) *M*=9 under the same out-of-plane compressive stress of 0.1 MPa. Contour diagrams of models with c) *M*=5 and d) *M*=9 under an out-of-plane shear stress of 0.1 MPa. All the RVE models have the same average graphene platelet diameter *d*_0_=500 nm, 100 complete platelets (N=100) in each layer, graphene area fraction *F_A_*=0.591, and graphene platelet regularity *α*=0.6.

Coupled effects of $\boldsymbol{\alpha}$ and $\boldsymbol{F}_{\boldsymbol{A}}$ on the elastic properties of MGPFs

**Figure S6**a shows that when the graphene platelet area fraction is $F_{A}=99.6\%$, the in-plane dimensionless Young’s modulus of MGPFs increases very slightly with the increase of the regularity degree $\alpha$ of the graphene platelets, and the Pearson correlation coefficient is $r=0.19546$ which indicates a weak positive dependence of $E_{1}$ on the graphene regularity degree $\alpha$. However, the magnitude variation of $E_{1}$ with $\alpha$ is very small and negligible. In addition, the magnitudes of all other independent elastic constants remain almost unchanged with the increase of the regularity degree $\alpha$, and the values of their Pearson correlation coefficient *r* are very close to 0. The results in Figure S6a suggest that if the mean size and the area fraction of the graphene platelets are sufficiently large, the graphene regularity degree has a negligible effect on the elastic properties of MGPFs. When the graphene platelet area fraction $F_{A}$ is fixed at $76.95\%$ or $59.17\%$, Figures S6b and S6c illustrate that the in-plane dimensionless Young’s modulus increases clearly with the increase of the regularity degree $\alpha$ of the graphene platelets, and the Pearson correlation coefficient *r* is $0.48356$ or $0.37785$, indicating a moderate positive dependence of $E_{1}$ on the regularity degree $\alpha$. All other elastic properties are almost independent of the regularity degree $\alpha$. Figures S6d-f show the geometric structures of graphene platelets in a single layer of the RVE model of MGPFs with $\alpha=0.6$ and different values of the graphene platelet area fraction $F_{A}$ .


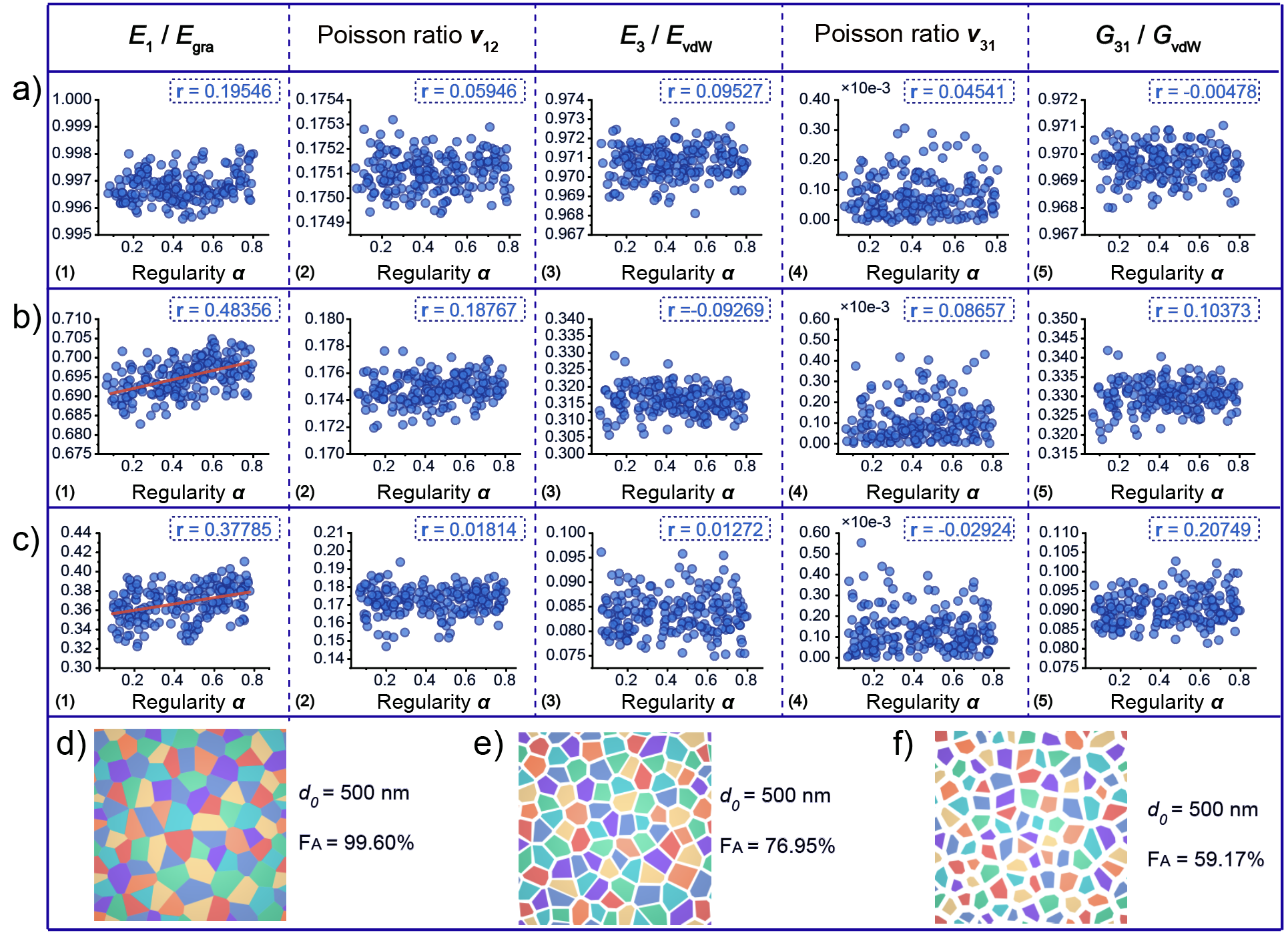


**Figure S6.** Coupled effects of the graphene platelet regularity degree $\alpha$ and area fraction $F_{A}$ on the dimensionless elastic properties of MGPFs with *M*=5 and $d_{0}=500$ nm. (a) graphene area fraction $F_{A}=99.6\%$; (b) graphene area fraction $F_{A}=76.95\%$; (c) graphene area fraction $F_{A}=59.17\%$; (d-f) geometric structures of graphene platelets in a single layer of the RVE models with $\alpha=0.6$, number of complete graphene platelets *N*=100, and different values of the graphene platelet area fraction $F_{A}$.

Comparison between the results in this work and those in literature

In the results of MGPFs obtained in this work, the in-plane Young’s modulus is normalized by that of the perfect graphene (i.e. $E_{gra}=$ 1000 GPa), the out-of-plane Young’s modulus and shear modulus are normalized by those of the equivalent isotropic solid layer material for van der Waals interaction (i.e. $E_{vdW}=$ 10 GPa and $G_{vdW}=$ 5 GPa), respectively. Thus, the dimensionless results obtained in this paper are applicable to similar single-material systems, such as multilayer graphene oxide and MXene, or other two-phase laminate composites composed of stiff and soft components, e.g. GO/Graphene-based composites and real nacre or seashell materials.

In order to compare the dimensionless in-plane and out-of-plane Young’s moduli of MGPFs obtained in this work with those of the relevant materials reported in literature, **Table S5** lists the material properties, geometric parameters and the dimensionless Young’s moduli of the relevant materials reported in literature. The detailed calculation about how to obtain the dimensionless in-plane and out-of-plane stiffnesses for the relevant laminate composites is described in the Applicability part of the main text.

**Table S5.** Summary of the elastic properties and geometric parameters of the components in the nacre-like layer-by-layer laminated composites reported in literature, and their dimensionless in-plane stiffnesses.

| Materials | Dimensionless  Modulus ^a)^ | E_com_ ^b)^ (GPa) | E_s_ (GPa) | t_s_ (nm) | E_m_ (GPa) | t_m_ (nm) | G_m_ (GPa) | d_0_ (μm) | l_0_ (nm) | Methods ^c)^ | Ref ^d)^ |
| --- | --- | --- | --- | --- | --- | --- | --- | --- | --- | --- | --- |
| GO | / | / | 175.7 | 0.34 | / | 0.7 | 0.173 | / | 7.77 | NC (2D) | [6] |
| GO | / | 44.63 | 175.7 | 0.34 | / | 0.7 | 0.173 | / | 7.77 | MD (2D) | [6] |
| MLG | / | / | 950 | 0.335 | / | 0.335 | 1 | / | 5.16 | MD (2D) | [7] |
| MLP | / | / | 105.5 | 0.554 | 4.01 | 0.554 | 0.96 | / | 2.90 | MD (2D) | [8] |
| Bio-Compos  (Φ=0.9) | 0.9102 | / | E_s_ | / | / | / | E_s_ /1000 | 37.68 | / | NC (2D) | [9] |
| Bio-compos  (Φ=20/21) | 0.7715-0.8620 | 73.48-82.1 | 100 | / | / | / | 4.6 | 7 × t_s_ | / | NC (2D) | [10] |
| Nacre-like  Composites | 0.7594 | / | 106 | 500 | 1.3 | 20 | 0.5 | 1-2 | 728.01 | FEM (3D) | [11] |
| Nacre  (Φ=0.95) | 0.6711 | 63.75 | 100 | 450 | 2.4 | / | 0.8 | 6 | 562.5 | FEM (2D) | [12] |
| Nacre(wet) | 0.7044 | 70 | 106 | 450 | ~2.8 | 30 | 0.8 | 8 | 668.72 | Exp +  FEM (3D) | [13] |
|  | 0.7813 (G_31_) | 10 (G_31_) |  |  |  |  |  |  |  |  | [13] |
| Nacre(wet) | 0.736-0.919 (E_3_) | 19.13 (E_3_) | 100-106 | 400-500 | 2.4±0.5 | 20-30 | 1 | 8 | 475±28 | Exp | [14] |
| Nacre(wet) | 0.788-0.876 (E_3_) | 19.7 (E_3_) | 100-106 | 400-500 | 2.4±0.5 | 20-30 | 1 | 1.8-7.7 | 475±28 | EXP | [15] |
| Nacre(wet) | 0.7446±0.08 (E_3_) | 29 (E_3_) | 79±15 | 435 | 2.84±0.3 | 35 | 1 | 8±0.2 | 493.56-598.15 | Exp | [16] |
| Nacre(wet) | 0.6191-0.7182 | 62.5-72.5 | 100-106 | 500 | / | 25 | 0.8-1.2 | 8 | 510.31-643.48 | Exp | [17] |
| MMT/PVA | 0.1830-0.3074 | 19.6 ± 1.0 | 178-270 | 1.28 | 1.4 | 2.12 | 0.2-0.6 | 0.119 | 14.19-24.57 | Exp | [18] |

^a)^ Dimensionless In-plane tensile modules of the whole material or model, if not explicitly stated.

^b)^ **Materials**: GO: graphene oxide, MLG: multilayer graphene, MLP: multilayer phosphorene assemblies, Bio-Compos: bio-composites, MTM: montmorillonite platelet, PVA: poly (vinyl alcohol). (Φ= t_s_/ (t_s_+t_m_ ))

^c)^ **Methods**: MD (Molecular dynamics), FEM (finite element methods), NC (Numerical calculation

^d)^ The references [6-18] cited here correspond to the references [54-66] cited in Figure 6, respectively.

References

[1] Z. Xue, G. Chen, C. Wang, R. Huang, *J. Mech. Phys. Solids* **2022**, *158*, 104698.

[2] E. Han, J. Yu, E. Annevelink, J. Son, D. A. Kang, K. Watanabe, T. Taniguchi, E. Ertekin, P. Y. Huang, A. M. Zande, *Nat. Mater.* **2020**, *19*, 305.

[3] C. Lee, X. Wei, J. W. Kysar, J. Hone, *Science* **2008**, *321*, 385.

[4] H. X. Zhu, J. R. Hobdell, A. H. Windle, *J. Mech. Phys. Solids* **2001**, *49*, 857.

[5] S. L. Omairey, P. D. Dunning, S. Sriramula, *Eng. Comput.* **2019**, *35*, 567.

[6] Z. He, Y. Zhu, J. Xia, H. Wu, *J. Mech. Phys. Solids* **2019**, *133*, 103706.

[7] W. Xia, L. Ruiz, N. M. Pugno, S. Keten, *Nanoscale* **2016**, *8*, 6456.

[8] N. Liu, J. Hong, X. Zeng, R. Pidaparti, X. Wang, *Phys. Chem. Chem. Phys.* **2017**, *19*, 13083.

[9] H. J. Lei, Z. Q. Zhang, F. Han, B. Liu, Y.-W. Zhang, H. J. Gao, *J. Appl. Mech.* **2013**, *80*, DOI 10.1115/1.4023976.

[10] S. Anup, *J. Mech. Behav. Biomed. Mater.* **2015**, *46*, 168.

[11] M. Maghsoudi-Ganjeh, L. Lin, X. Yang, X. Zeng, *J. Mater. Res.* **2021**, *36*, 2651.

[12] F. Barthelat, *J. Mech. Phys. Solids* **2014**, *73*, 22.

[13] F. Barthelat, H. Tang, P. D. Zavattieri, C.-M. Li, H. D. Espinosa, *J. Mech. Phys. Solids* **2007**, *55*, 306.

[14] R. Menig, M. H. Meyers, M. A. Meyers, K. S. Vecchio, *Acta Mater.* **2000**, *48*, 2383.

[15] D. Jiao, Z. Q. Liu, Y. K. Zhu, Z. Y. Weng, Z. F. Zhang, *Mater. Sci. Eng.: C* **2016**, *68*, 9.

[16] F. Barthelat, C.-M. Li, C. Comi, H. D. Espinosa, *J. Mater. Res.* **2006**, *21*, 1977.

[17] F. Song, J. Zhou, X. Xu, Y. Xu, Y. Bai, *Phys. Rev. Lett.* **2008**, *100*, 245502.

[18] J. Wang, Q. Cheng, L. Lin, L. Jiang, *ACS Nano* **2014**, *8*, 2739.
